# Temporal Dynamics of Latent Fingerprint Microbiomes: A First Step to Decoding Crime Evidence

**DOI:** 10.3390/genes17080931

**Published:** 2026-08-10

**Authors:** Josep De Alcaraz-Fossoul, Samantha J. Sawyer

**Affiliations:** Forensic Science Department, Henry C. Lee College of Criminal Justice and Forensic Science, University of New Haven, West Haven, CT 06516, USA; ssawyer@newhaven.edu

**Keywords:** microbiome, fingerprint, time, microbe, morphometric, profiling, imaging, time since deposition, aging, degradation

## Abstract

Background/Objectives: Latent fingerprints (LFs) have been a cornerstone of forensic identification through conventional friction ridge pattern analysis; however, the microbial communities they harbor remain a largely untapped source of information. Estimation of the time-since-deposition (TsDp) of LFs is still an unresolved challenge in forensic science, as existing 2D and 3D imaging methodologies provide limited temporal resolution. This study investigated whether temporal shifts in LF-associated microbiota could potentially complement dating approaches via genetic analyses. Methods: LFs were collected from two healthy donors from both hands, pre- and post-hand washing, across three time points spanning 192 h (8 days) under monitored, but uncontrolled, indoor conditions. LF friction ridges were optically examined via 2D and 3D imaging, while microbial communities were characterized by *16S rRNA* gene sequencing targeting the V3-V4 region. Microbial profiles were analyzed to distinguish temporally stable core microbiota from transient taxa (alpha and beta diversity) and to statistically identify microbial panels associated with donor, handedness, hand-washing status, and/or temporal succession. Results: A stable core microbiota, dominated by *Actinobacteria* and *Bacilli*, persisted across donors, handedness, hand-washing status, and time points. Transient low-abundance taxa, including *Nitriliruptoria* and *Phycisphaerae*, exhibited temporal fluctuations influenced by donor characteristics, hand-washing status, and post-deposition interval. Upon excluding time-invariant stable taxa, donor-specific biological profiles comprising 32 and 28 class-rank taxa were selected for each donor, revealing individualized patterns of microbial dynamics. Conclusions: LF-associated microbiomes contain both stable and temporally dynamic taxa, with the potential to provide personalized biological information for TsDp estimation. These preliminary findings contribute to the molecular toolkit of forensic microbiomics by laying the foundation for prospective multimodal models integrating LF microbial and topographical data to improve the temporal interpretation of crime evidence touched by bare hands. Further validation with broader donor cohorts and environmental conditions are essential before operational forensic implementation.

## 1. Introduction

Latent fingerprints (LFs), also referred to as fingermarks [1,2], are complex three-dimensional (3D) “living” structures composed of a mixture of endogenous secretions and exogenous compounds [3,4,5,6,7,8,9,10,11,12,13]; they harbor a diverse microbial community that is directly transferred from the donor’s skin upon contact with a surface [14,15,16,17,18,19]. If undisturbed, the chemistry and topography of friction ridges undergo measurable alterations over time that can be recorded via several chemical and optical methods [4,10,11,20,21,22,23,24,25,26,27]. Among the optical methods, 2D and 3D imaging technologies have introduced and evaluated several morphometrics as suitable approaches to potentially estimate time-since-deposition (TsDp) (or the “age”) of LFs. However, these technologies are limited in scope and/or practical field applications [4]. The physicochemical degradation of friction ridges is primarily driven by the interaction of (a) environmental conditions, such as temperature, relative humidity, and degree of natural light exposure; (b) surface properties, such as porosity, roughness, and wettability; and (c) donor characteristics, such as biological sex, levels of perspiration, and friction ridge morphology [4,11,20,21]. Likewise, the microbial communities associated with LFs are expected to influence friction ridge degradation as well as be shaped by the combined effects of these variables [28,29,30,31,32,33,34,35]. However, the composition, temporal dynamics, and forensic relevance of the LF-associated microbiome remain poorly characterized.

The dynamic nature of the “touch microbiome” [36] provides a promising biological source of forensic information that could become valuable for estimating TsDp, a distinct application to source (donor) identification [14,16,17,19,30,31,32,33,34]. Although the latter represents an emerging area of forensic research, it falls beyond the scope of the present study, as the microbial profile of an LF may diverge from that of the donor’s skin within hours after deposition, mostly due to microbial succession and environmental influences [17,29]. On the other hand, these temporal changes within the LF microbiota may provide greater discriminatory power than conventional optical and/or chemical approaches for estimating TsDp [4].

Over the past decade, attention has been directed to elucidate how certain variables, including skin flora, environmental exposure, and personal hygiene, shape the human skin microbiome [31,35,37,38]. Indeed, recent advances in molecular sequencing technologies have enabled the application of microbial profiling to common forensic challenges, including donor identification [14,26,32,33], source attribution of bodily fluids [19,39], postmortem interval (PMI) estimation [40,41,42], and geolocation inference [19,40,43]. Other studies [29,39] have also demonstrated that microbial communities within LFs undergo measurable temporal succession following deposition [29,39], supporting the hypothesis that certain LF-associated microbes (i.e., time-dependent microbial profiles) may provide valuable forensic intelligence for crime scene reconstruction.

Despite these advances, the effect of factors such as handedness, hand washing, and the elapsed time post-deposition on the abundance, occurrence, composition, and succession of the “touch microbiome” via *16S rRNA* gene sequencing remains poorly characterized [29,36]. This knowledge gap limits the potential application of LFs as a basis of genetic microbial profiling for dating touched evidence based on the transferred microbiota from friction ridge skin. In fact, only a limited number of studies have specifically investigated the potential of LF-associated microbes as biological indicators for TsDp estimations [29,34,36]. This emerging concept, referred to as the *chronomicrobiome* [29], proposes that predictable temporal changes in select microbial communities may function as “biological clocks” capable of dating LFs and, by extension, other items of physical evidence handled with bare hands.

Reliable estimation of TsDp will require a comprehensive understanding of the initial biological, chemical, and topographical characteristics of LFs, as well as how these features evolve after deposition. A plethora of donor- and environment-related factors may affect both the microbial communities transferred and their subsequent temporal succession. These may encompass a wide variety of variables, from the type of receiving surface [44] to the duration and pressure of contact between the friction ridges and the substrate [45]. The present study focuses on three unexplored parameters: (i) handedness (dominant vs. non-dominant hand); (ii) hand-washing status (pre- vs. post-washing); and (iii) post-deposition interval (ranging from 6 to 192 h). Specifically, it investigates the effects that these factors have on microbial occurrence, composition, and succession within LFs deposited by two unrelated individuals in a monitored, yet uncontrolled, dark indoor location, with a secondary focus on relative taxonomic abundances.

The current proof-of-concept experiment was precisely designed to emulate a pseudo-realistic forensic scenario in which LFs are recovered from handled objects in a criminal event, such as a weapon (e.g., knife or firearm) allegedly involved in a homicide inside a home. In situations where friction ridge detail may be insufficient for conventional identification—or where no reliable temporal indicators are present—and multiple contributors have handled the same object, time-variant LF-associated microbial profiles could help establish the chronological order of placement (i.e., TsDp), thereby supporting event reconstruction and the interpretation of contributor involvement.

To further explore the interplay between microbial succession and LF degradation, one 2D morphometric (2D-BG) [46,47] and one 3D topographical metric (3D-Sa) [22,47] were evaluated alongside microbial community profiling [48]. The integration of optical, topographical, and microbiological analyses provides a complementary multimodal framework for elucidating TsDp and establishes the foundation for developing future predictive models for estimating the age of LFs.

## 2. Materials and Methods

### 2.1. Latent Fingermark Deposition and Aging Process

Upon approval from the Institutional Review Board (IRB), two healthy donors with no skin conditions (one left-handed female and one right-handed male) were selected. They refrained from drinking alcohol and caffeine and applying creams or lotions for twelve hours prior to deposition, as well as taking antibiotics for at least one week. Glass microscope slides were cleaned with 100% *v*/*v* bleach, 70% *v*/*v* ethanol, and distilled water. These were placed on an electronic scale to measure pressure during the latent fingerprint (LF) deposition process, typically between 400–1000 g. The two donors deposited LFs from the thumb and index finger from their dominant and non-dominant hands in triplicate on the same day to be examined at fresh (T1 = 3 to 6 h), T96 = 96 h, and T192 = 192 h post-deposition time points (totaling 240 for 3D analyses and 144 for 2D analyses). For the pre-washed hand condition, donors washed their hands with soap and water two hours before arriving in the laboratory and went about their routine, refraining from participating in activities that would require them to wash their hands again, such as eating or shaking hands with others. The donors then tapped and rubbed the oily and sebaceous areas of their face with the tips of their fingers to mimic real scenarios before the imprinting process [20]. For the post-washed condition, donors were asked to wash their hands with soap and water, but, in this case, they “recharged” their fingers by touching their face between every deposition without any significant waiting time. These were prepared in three sets: one for 2D imaging, one for 3D imaging, and one for microbial analysis. Then, the specimens for 2D and 3D examinations were stored in the dark at room temperature to age until the next time point. The temperature (°C) and relative humidity (%RH) were automatically recorded throughout the experiment with an EasyLog (EL-USB-2-LCD RH/Temp) thermohygrometer (Lascar Inc, Dayton, OH, USA). Data were collected every 15 min.

### 2.2. Topographical Examinations of LFs by 2D and 3D Imaging

In order to determine the level of integrity of dactylograms (friction ridge patterns), the 2D-imaging set (72 LFs per donor = 2 fingers × 2 hands × 2 washing conditions × 3 time points × 3 replicas) was visualized with titanium dioxide powder (white color) and photographed at each time point with a Canon EOS Rebel T5i digital camera (Tokyo, Japan), following a previously described protocol [20]. One morphometric was collected for this analysis, *BlueGreen* (2D-BG) [46,47] for ridge clarity, where a color map was automatically overlaid on the dactylogram, as blue and green colors present the highest qualities on a scale of four colors, including yellow and red (the lowest). The 3D-imaging set (120 LFs per donor = 2 fingers × 2 hands × 2 washing conditions × 3 time points × (6 replicas T1 + 6 replicas T96 + 3 replicas T192)) was scanned on the Optical Profilometer as described in the literature [22] with Zygo Corporation’s ZeGage^TM^ Pro (Middlefield, CT, USA). The 3D-*Sa* metric (average roughness of surface) was collected to measure the average heights of friction ridges [22]. These imaging processes were a preliminary checkpoint to verify the presence of viable ridge impressions; thus, the data are not widely discussed.

### 2.3. DNA Collection and Extraction

At each time point, glass microscope slides bearing a microbiome set were transferred to a −80 °C freezer until DNA collection. All LFs were removed from the freezer and left to thaw, and DNA from the thumb and index finger was randomly collected with the same cotton swab (Dealmed Sterile Cotton-Tipped Wood Applicators) by pooling DNA from both finger impressions of the same hand, representing the microbial community associated with one hand. Interfinger variation was not a factor of interest for microbial analysis. Negative controls (i.e., swabs of empty slides) and positive controls (i.e., direct finger swabs) were also prepared. The microbial communities were characterized by sequencing the *16S rRNA* gene (V3-V4 region), and occurrence and relative abundances were compared across conditions. A modified protocol of the Qiagen DNeasy Blood & Tissue Kit (Germantown, MD, USA) was adapted for DNA extraction. This included a wet/dry swab method with a lysis buffer. The swab tip was placed in a single collection tube for extraction. The solution was transferred to a collection column, and the remainder of the extraction protocol was followed, with a final elution volume adjusted to 35 µL. The ThermoScientific NanoDrop One/OneC Spectrophotometer (Waltham, MA, USA) quantified DNA before *16S rRNA* gene sequencing by Zymo Research, Irvine, CA, USA.

### 2.4. Microbial DNA Library Preparation and Sequencing

A select number of representative (random) duplicate LF samples (12 per donor, 4 positive controls, and 2 negative controls) were processed and analyzed with the ZymoBIOMICS^®^ Targeted Sequencing Service (Zymo Research, Irvine, CA, USA). Bacterial *16S rRNA* gene sequencing was performed with the Quick-16S™ NGS Library Prep Kit (Zymo Research, Irvine, CA, USA). The primers amplified the V3-V4 region and were custom-designed by Zymo Research. The sequencing library was prepared using real-time PCR, and the final products were quantified with qPCR fluorescence readings. The final pooled library was cleaned with the Select-a-Size DNA Clean & Concentrator™ (Zymo Research, Irvine, CA, USA), then quantified with TapeStation^®^ (Agilent Technologies, Santa Clara, CA, USA) and Qubit^®^ (Thermo Fisher Scientific, Waltham, MA, USA). The ZymoBIOMICS^®^ Microbial Community DNA Standard (Zymo Research, Irvine, CA, USA) was used as a positive control for each targeted library preparation. Negative controls (i.e., blank extraction control, blank library preparation control) were included. The final library was sequenced on Illumina^®^ Nextseq™ (San Diego, CA, USA) with a P1 reagent kit (600 cycles). The sequencing was performed with a 30% PhiX spike-in. Unique amplicon sequence variants were inferred from raw reads using the DADA2 pipeline. Potential sequencing errors and chimeric sequences were also removed with the DADA2 pipeline. Taxonomy assignment was performed using Uclust from Qiime v.1.9.1 with the Zymo Research Database. Composition visualization and alpha-diversity analyses were performed with Qiime v.1.9.1. No failed samples were reported.

### 2.5. Microbial DNA Quantification

A quantitative real-time PCR was performed by the external provider (Zymo Research, Irvine, CA, USA) and set up with a standard curve made with plasmid DNA containing one copy of the 16S gene prepared in 10-fold serial dilutions. The primers were the same as those employed in Targeted Library Preparation. The equation generated calculated the number of gene copies per microliter in each DNA sample and was obtained by dividing the gene copy number by an assumed number of gene copies per genome. The value for 16S copies per genome is 4. The quantity of DNA per microliter DNA sample (DNA_ng) was calculated assuming a genome size of 4.64 × 106 bp, the genome size of *Escherichia coli*. The values obtained were therefore approximate quantitative estimates rather than absolute measures of microbial biomass.

### 2.6. Microbial Composition Statistical Analysis

Microsoft^®^ Excel^®^ and IBM^®^ SPSS^®^ Statistics 25 were used to perform statistical analysis. The 3D-*Sa* raw data were normalized with Microsoft^®^ Excel^®^ as described previously [45] and formatted for further analysis in IBM^®^ SPSS^®^. To understand the distribution of data for different factors, box plots were prepared. Normality and homogeneity of variance tests were performed in IMB^®^ SPSS^®^ for the 2D, 3D, and microbial data. One-way ANOVA, univariate analyses, and post hoc Tukey tests were initially performed to reveal the relationship between factors and how they influence one another (data available upon request). The nonparametric Kruskal–Wallis test examined differences between factors and stratified combinations. Statistical significance was ascribed to an alpha level of the adjusted *p*-values ≤ 0.05. Eta-squared effect sizes (η^2^H) were calculated for Kruskal–Wallis analyses with the H statistic, degrees of freedom, and sample size. Negative η^2^H values were interpreted as zero [49]. Stratified Kruskal–Wallis analyses evaluated factor-specific effects within donor, time-since-deposition, hand-dominance, and hand-washing condition subsets. The recommended guidelines [49] for effect sizes were as follows: small effect = 0.01 to <0.06; moderate effect = 0.06 to <0.14; and large effect = 0.14 or higher. These analyses were interpreted as exploratory stratified comparisons rather than formal factorial interaction tests. Analysis of sequenced DNA samples was performed with QIIME 2™. Taxonomy graphs (alpha diversity) were generated for different factors, and the relative microbial abundance of the samples was assessed to generate a heat map of microbial succession over time in Microsoft^®^ Excel^®^.

Donor-specific “microbial panels” or “profiles” were generated with predefined filtering criteria. Temporally stable core taxa were excluded, and only taxa with >50 sequencing reads and temporal variation across the three time points were retained. For duplicate samples, a taxon was considered present if detected in at least one replicate, and in the case of having two reads, the higher read count was taken for analysis. These panels represented exploratory candidate taxa for temporal profiling rather than validated biomarkers.

To evaluate whether LF microbial communities changed according to donor, time since deposition, hand dominance, and hand-washing status, microbial community analyses were performed in R (R Core Team, version 4.4.1, *Race for Your Life*) using the packages *vegan*, *ggplot2*, *dplyr*, *tidyr*, and *ggrepel*. To minimize bias arising from differences in sequencing depth and the disproportionate influence of highly abundant taxa, amplicon sequence variant (ASV) counts were converted to relative abundances by standardizing each sample to unit total abundance. This normalization enabled comparisons of microbial community composition across samples while reducing the influence of unequal sequencing depth and dominant taxa.

Microbial community composition (β-diversity) was quantified with the Bray–Curtis dissimilarity index (DI), which ranges from 0 (identical communities) to 1 (completely dissimilar communities). Temporal community turnover was assessed by calculating pairwise Bray–Curtis dissimilarities between consecutive sampling intervals (T1–T96, T96–T192) and across the entire aging period (T1–T192) for each donor, hand-dominance, and hand-washing condition. Higher Bray–Curtis values indicated greater changes in microbial community composition through time and were interpreted as evidence of increased ecological succession. To assess the persistence of donor-associated microbial panels, Bray–Curtis dissimilarities were also calculated between donors while controlling for hand dominance, hand-washing status, and sampling time. Comparisons were performed at equivalent deposition intervals across treatments to determine whether donor microbial communities remained distinguishable throughout the aging period.

Microbial community assemblages were visualized among donors, time intervals, handedness, and hand-washing treatments with Principal Coordinates Analysis (PCoA) based on Bray–Curtis dissimilarity matrices. Samples were projected onto the first two principal coordinate axes to facilitate visualization of community clustering and temporal changes in community composition.

Community trajectories were quantified to characterize the magnitude and direction of microbial succession within each donor, handedness, and hand-washing condition combination across time using calculated Euclidean distances traveled between consecutive time points in ordination space. To determine whether community succession followed a more directional/linear path over a more complex and unpredictable trajectory, the proportion of net change (*E*_1,3_) and trajectory length (*E*_1,2_ + *E*_2,3_) of each treatment combination was compared using the following equation:

(1) $$Directional\;Index=\frac{E_{1,3}}{E_{1,2}+E_{2,3}}$$

where *E*_1,2_ represents the Euclidian distance between the sample at T1 and T96, *E*_2,3_ represents the Euclidian distance between T96 and T192, and *E*_1,3_ represents the Euclidian distance between T1 and T192, for a respective treatment. Indices approaching 1 indicate a directional or linear succession, and lower values indicate a more complex community succession through ordination space. Prior to conducting the analysis, to cleanly categorize samples for descriptive comparison within this dataset, the following categories were arbitrarily established: >0.70 is “Highly Directional”, <0.40 is “Complex” or “Wandering”, and values between these categories (0.40–0.70) are “Intermediate”.

### 2.7. Exploratory Analysis to Identify Temporal Taxa

To assess whether any taxonomic groups could be uniquely associated with a time point or by donor, relative abundances were averaged within each collection time (T1, T96, T192) and within each donor separately. In addition to comparing the most abundant taxa, taxon association was calculated through a specificity metric using the following equation:

(2) $$Specificity=\frac{Maximum\;Mean\;Relative\;Abundance}{Sum\;of\;Mean\;Relative\;Abundances}$$

where specificity values approaching 1 indicate strong association with a single time point or donor.

## 3. Results

### 3.1. Morphometric Examination of LF Friction Ridges Between Conditions

**Figure 1:**
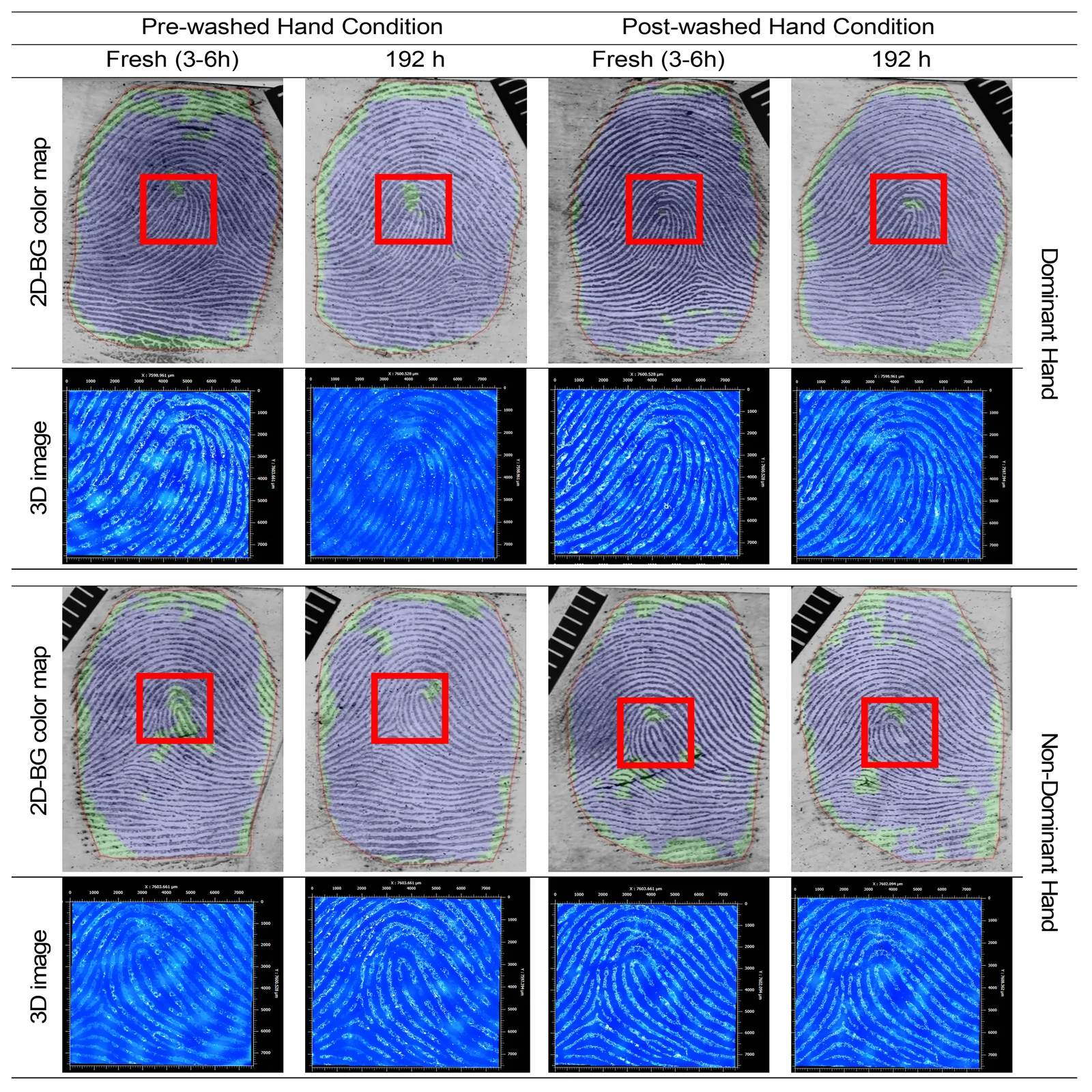
Examples of 2D-BG color-coded and 3D (flattened) images depicting differences between hand-washing status and handedness over time. These images are from the female’s LFs (thumbs) and only show the starting and ending time points. Note that the color of ridges for 2D-BG is inverted from the white-powdered original. In these images, the extension of blue and green does not change significantly. The red square represents the area scanned with the OP to obtain 3D scans. The colors in these images are the selection of the examiner, where whiter intensities represent taller ridge areas.

The indoor environment (18.0–24.5 °C, mean 20.6 °C; relative humidity 31–90%, mean 66.4%) led only to minor changes in LF ridge morphology throughout the 192 h aging period. A simple visual inspection revealed minimal deterioration in ridge detail irrespective of donor, handedness, hand-washing status, and post-deposition interval (Figure 1). A more detailed quantitative assessment of ridge degradation with the 2D-BG [45] and 3D-*Sa* morphometrics [22] partially supported these initial qualitative observations while revealing subtle, otherwise imperceptible alterations (Supplemental Tables S1–S3 and Figures S1 and S2).

The metric 2D-BG (Supplemental Table S1) revealed a significant donor effect (*p* = 0.006), indicating that ridge degradation patterns may differ between the two individuals and that subsequent donor-specific inferences were preferred. For instance, when donors were analyzed separately (Supplemental Table S3), hand washing and post-deposition interval significantly influenced ridge clarity in both individuals (*p* < 0.05), whereas handedness affected only the male (*p* = 0.002). More specifically, the effect of handedness was detectable exclusively in pre-washed LFs from both donors (*p* < 0.005), while temporal differences were observed exclusively in the non-dominant (left) hand of the male and the dominant (left) hand of the female. In contrast, 3D-*Sa* detected no significant effects of donor, handedness, hand-washing status, or elapsed time (Supplemental Tables S1 and S2), indicating that average ridge heights remained largely unchanged across conditions and thus were not sufficiently informative for TsDp estimation purposes. Taking them together, these results highlight the limited temporal sensitivity of current optical morphometric approaches for short-term LF age determinations and emphasize the need for complementary genetic biomarkers capable of distinguishing donors and post-deposition intervals with greater accuracy.

### 3.2. Variability of Core Taxa Across Conditions: Temporal Dynamics

**Figure 2:**
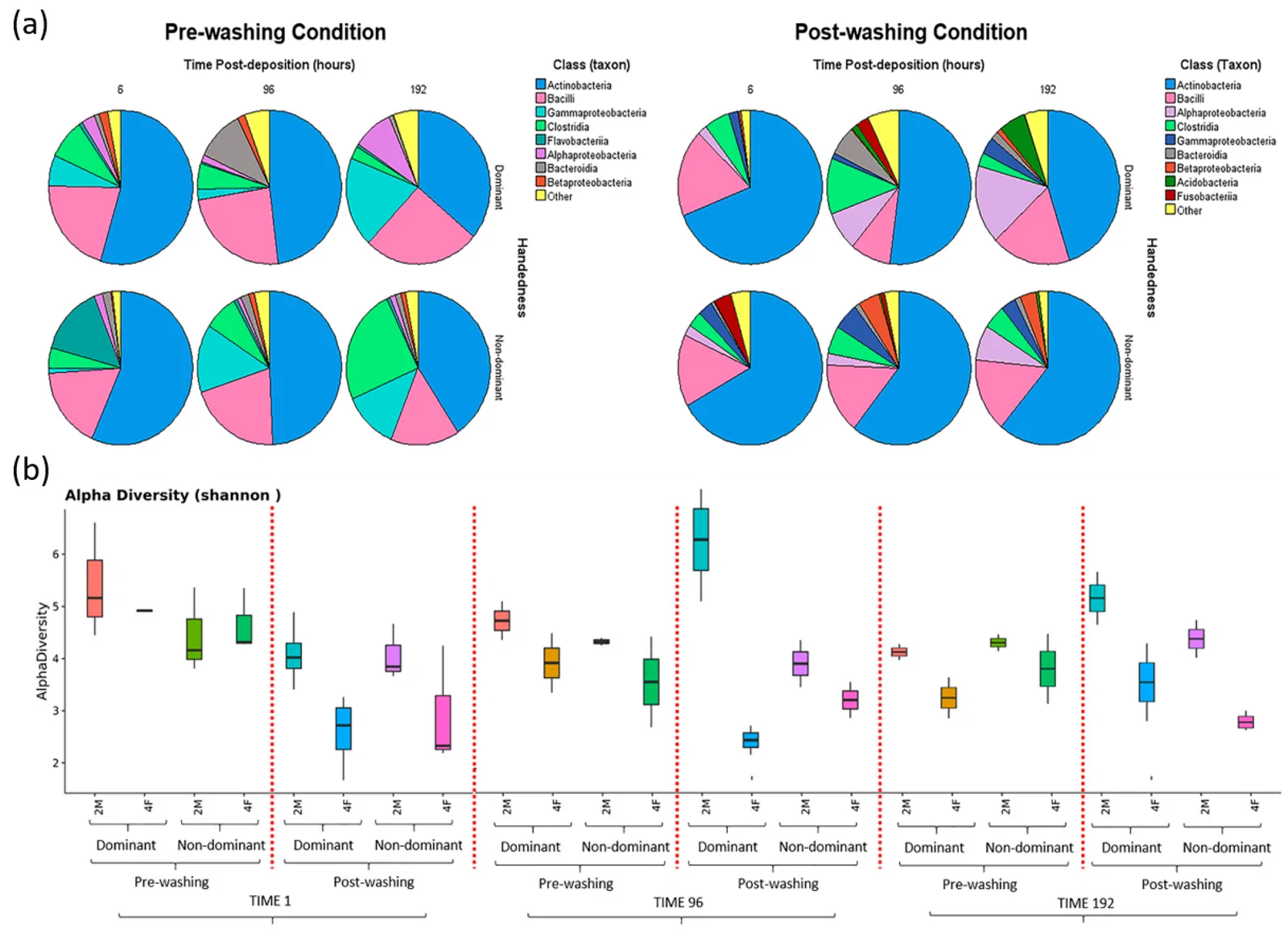
Pie charts and alpha diversity boxplots of LF-associated microbiota reveal ubiquitous taxa and changes in diversity by hand-washing conditions and handedness over time. (**a**) Note the ubiquitousness and high abundance of *Actinobacteria* and *Bacilli* across all conditions. The yellow slice, labeled as “other”, represents the primary focus of the current study, the highly dynamic time-variant taxa. These charts combine microbiota from both individuals at the class taxonomic rank. (**b**) Alpha diversity plots split by male (2M) and female donor (4F) depicting the combined effect of time, hand washing, and handedness. Data are from the observed species in each sample per time point (T1 = 3–6 h; T96 = 96 h; T192 = 192 h post-deposition). Each boxplot includes data from n = 2.

Taxonomic analysis of LF-associated microbiota identified seven dominant classes, including *Actinobacteria*, *Alphaproteobacteria*, *Bacilli*, *Bacteroidia*, *Betaproteobacteria*, *Clostridia*, and *Gammaproteobacteria*, that remained highly abundant across all experimental conditions: donor, handedness, hand-washing status, and post-deposition interval (Figure 2a and Supplemental Figures S3 and S4). These taxa accounted for more than 95% of the microbial community in nearly all samples, with only modest fluctuations in relative abundance, primarily associated with handedness (Supplemental Table S4). Among them, *Actinobacteria* and *Bacilli* were the predominant classes throughout the study.

Although the overall composition of the LF core microbiota remained remarkably stable, a few statistically significant differences were observed. Hand washing significantly affected the dominant hand at the earliest sampling point (T1, 3–6 h post-deposition; *p* = 0.004), as shown in Supplemental Table S5. The effect of handedness was rarely detected, except for the male at T96 (96 h post-deposition; *p* = 0.001), which may be mostly influenced by the effect of elapsed time rather than hand dominance, as this difference was not noticed on freshly deposited LFs (T1; *p* = 0.491). A significant temporal effect was detected only for post-washed dominant hands (*p* < 0.001). These data suggested that the dominant microbial classes experienced limited temporal variation over the 192 h aging period; thus, their utility as temporal biomarkers for estimating TsDp would be inadequate. Therefore, subsequent analyses focused on the less abundant transient taxa to assess whether these microbial populations showed larger temporal sensitivity and could be better indicators of LF aging (Table 1).

The overall effects of handedness, hand-washing status, and elapsed time were negligible in the combined donor analysis (Table 1). When the factor of time was stratified by handedness and hand-washing condition, the largest effects were observed only in the dominant hand after washing, both for abundance (η^2^ = 0.035; *p* = 0.008) and occurrence (η^2^ = 0.026; *p* = 0.021), mostly after 96 h post-deposition. However, donor-stratified analyses revealed distinct patterns. For the male, time was the most significant factor, but with small effects for abundance (η^2^ = 0.020, *p* = 0.007) and occurrence (η^2^ = 0.016, *p* = 0.016). Once stratified, the strongest effects for this donor were observed with washed hands at 96 h post-deposition for occurrence (η^2^ = 0.157; *p* < 0.001) and abundance (η^2^ = 0.124; *p* = 0.002). Time effects were also strongest in the dominant hand after washing hands, for abundance (η^2^ = 0.170) and occurrence (η^2^ = 0.155), both *p* < 0.001. For the female, time was again the only significant main factor, with η^2^ = 0.024 for abundance (*p* = 0.004) and η^2^ = 0.034 for occurrence (*p* < 0.001). The strongest effects were observed for time in the non-dominant hand, particularly in post-washed conditions, where η^2^ = 0.062 for abundance (*p* = 0.013) and η^2^ = 0.072 for occurrence (*p* = 0.008). Hand-washing status showed negligible effects for this donor across stratified analyses. Together, these results suggest that time-dependent microbial changes were donor-specific and most evident in post-washed hand samples.

The impact of donor, hand dominance, hand-washing status, and time since deposition on LF-associated microbial communities (i.e., alpha diversity) was assessed with the Shannon diversity index, which measures taxonomic richness and community evenness while remaining sensitive to low-abundance taxa (Figure 2b). Hand washing had the most pronounced effect on microbial diversity immediately after deposition (T1), with pre-washed LFs consistently showing higher diversity than post-washed samples. Minor differences associated with handedness were observed in pre-washed LFs but became negligible following hand washing, indicating that hygiene practices had a stronger influence on community diversity than handedness. This pattern was consistent for both donors, agreeing with previous studies on the effect of this factor on skin microbial diversity and composition [31].

Although microbial diversity declined immediately after hand washing, the temporal trajectories differed between donors. For the male, diversity increased in post-washed dominant hands by T96, whereas by T192 diversity had slightly decreased. In contrast, the female exhibited a more persistent reduction in diversity following hand washing. Throughout the aging period, pre-washed samples maintained relatively high and stable diversity, showing only a gradual decline, whereas post-washed communities generally stabilized or showed a slight recovery over time.

These results indicated that, despite the compositional stability of the dominant LF core microbiota, the overall community diversity changed over time and was strongly influenced by hand washing and, to a lesser extent, by source composition (i.e., donor microbial identity) and post-deposition interval. The small temporal variation detected among dominant taxa may suggest that microbial succession is driven primarily by low-abundance (transient) microbes, providing the rationale for examining transient taxa as more time-sensitive biomarkers for LF aging and TsDp estimations.

### 3.3. Variability of Transient Taxa Across Conditions: Temporal Dynamics

**Figure 3:**
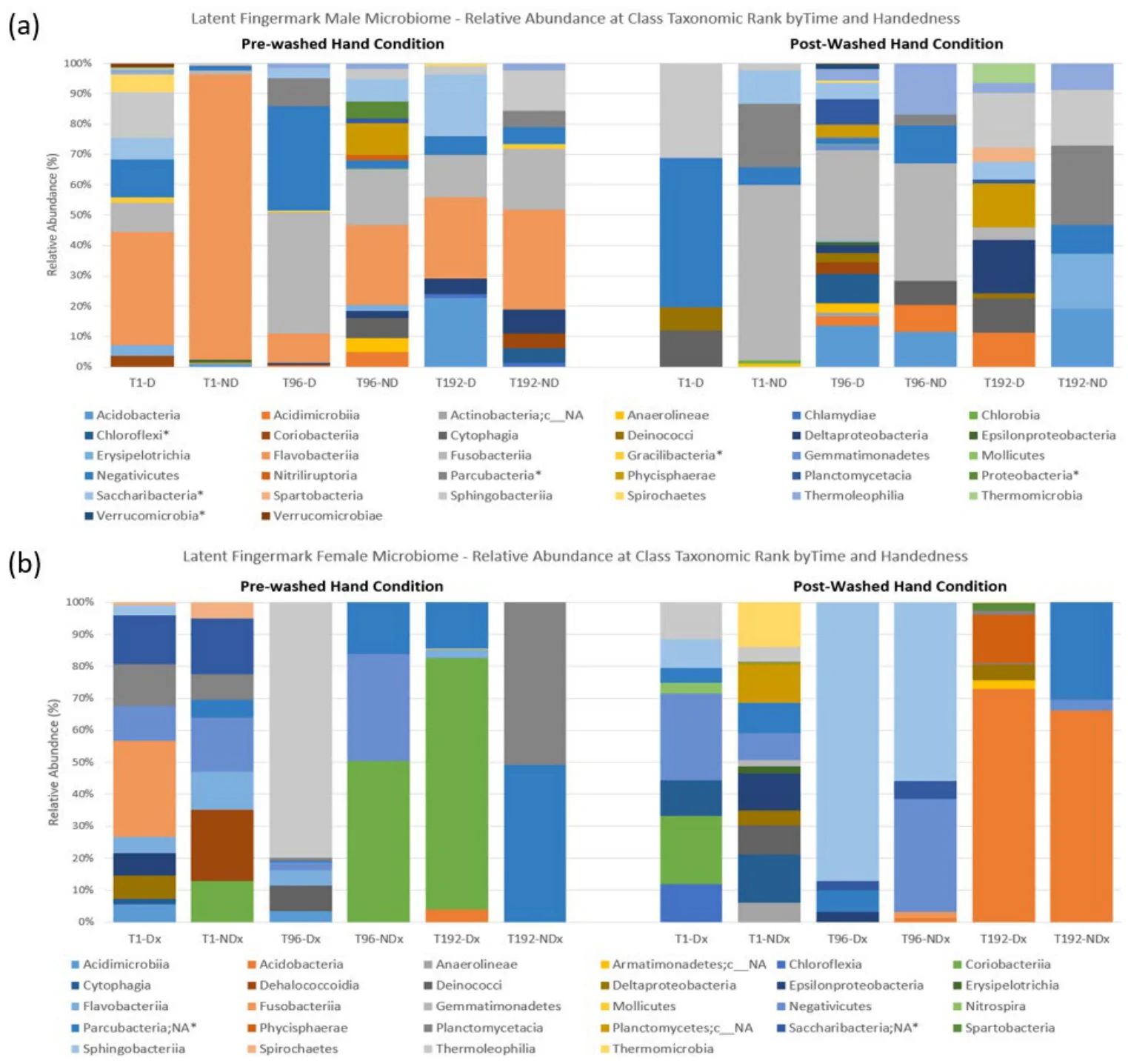
Microbial profiles selected from the pool of low-abundant and transient taxa reveal distinct population compositions by hand-washing status and handedness over time for each donor. (**a**) Relative abundance of male LF microbial composition over time (T1 = 3–6 h; T96 = 96 h; T192 = 192 h), showing dominant (D) and non-dominant (ND) hands per hand-washing condition. (**b**) Relative abundance of female LF microbial composition. NA means “Not Assigned”, in which the database could not match a DNA sequence or organism to a specific taxonomic rank. (*) represents taxa detected in one donor only. The occurrence of taxa is considered more relevant than the relative abundance for the purpose of developing time-dependent microbial panels.

**Figure 4:**
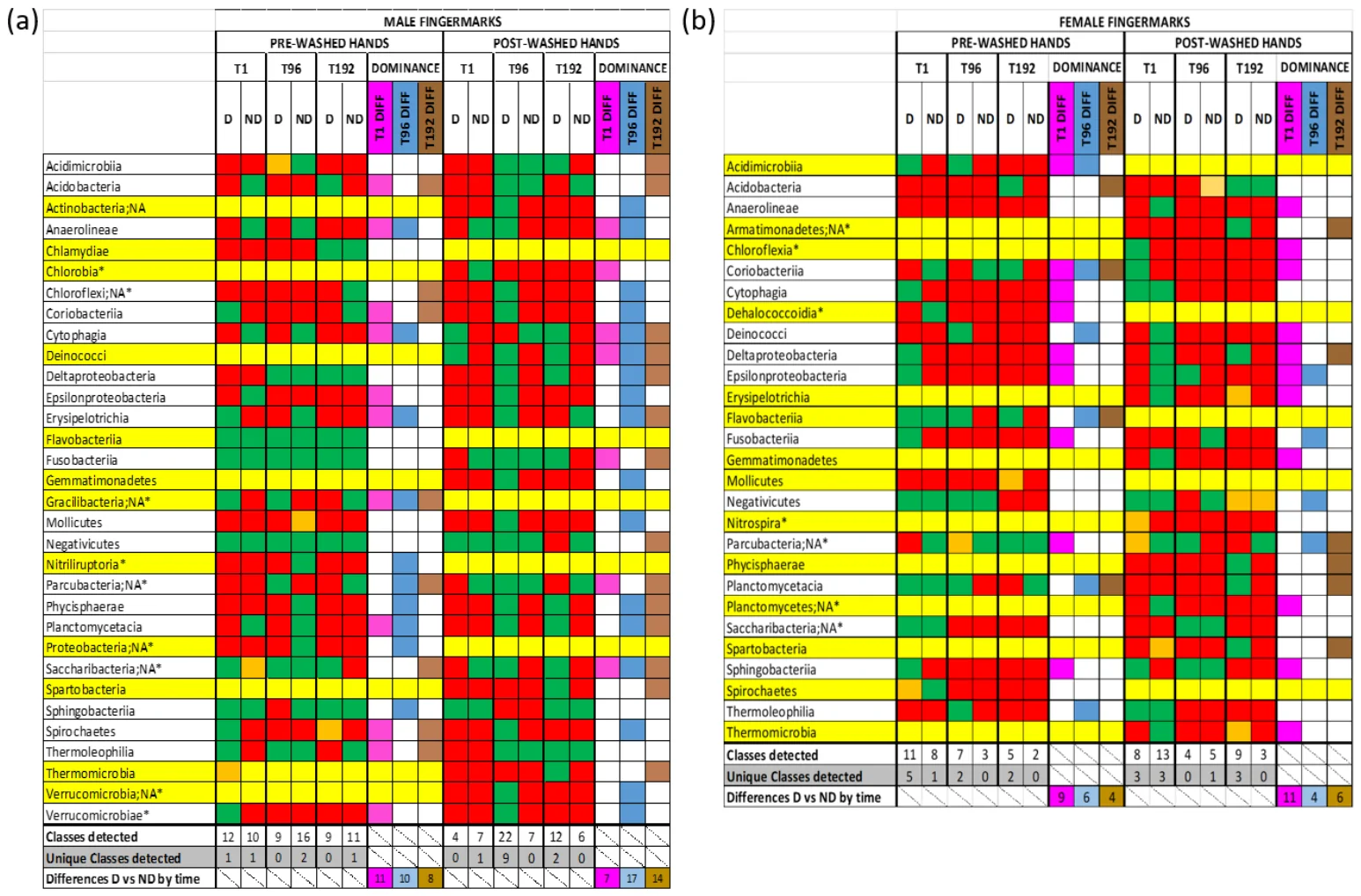
Microbial panels selected from the pool of low-abundant and transient taxa reveal the occurrence of taxa over time for each donor. (**a**) Presence (in green) and absence (in red) of male LF microbial taxa over time (T1 = 3–6 h; T96 = 96 h; T192 = 192 h), showing dominant (D) and non-dominant (ND) hands per hand-washing condition. (**b**) Results for the female. (*) represents taxa detected in one donor only. Orange designates potential presence due to very low relative abundance (10 to 50 reads) and/or no detection in duplicates. The yellow highlights differences between pre- and post-hand washing. The “dominance” columns highlight the instances where handedness played a distinctive role.

To increase temporal resolution and better characterize microbial succession, subsequent analyses focused on low-abundance, time-variant taxa (<10% relative abundance) after excluding the seven dominant, temporally stable classes previously identified as the core LF microbiota (Figure 3 and Figure 4). This strategy was adopted to enhance the detection of subtle temporal changes that could otherwise be masked by the highly abundant core community and was inspired by other forensic microbiome studies demonstrating predictable microbial succession for estimating PMI [40,41,42].

Following removal of the core microbiota, donor-specific microbial profiles were generated independently (Figure 3 and Figure 4 and Table 1). These were based on the occurrence (presence/absence) of transient taxa across post-deposition intervals, handedness, and hand-washing status. A total of 32 class-rank taxa were retained for the male, and 28 for the female. Although additional refinement is possible, these taxa represented possible microbial candidates capable of capturing individualized temporal changes in LF microbiota with no intent to be generalizable to a population of donors.

From the examined variables, hand washing produced the greatest immediate effect on transient LF microbial communities (Figure 3). At the earliest sampling time (T1), pre-washed LFs consistently displayed greater microbial diversity than post-washed samples, which showed comparatively more homogeneous community compositions. As aging progressed, microbial communities from washed hands became increasingly diverse, gradually diverging from their initial donor-associated composition. Several transient taxa were linked with specific experimental conditions. For example, *Nitriliruptoria* and *Flavobacteriia* were detected exclusively in pre-washed samples, whereas *Spartobacteria* and *Verrucomicrobiae* occurred only following hand washing.

Handedness also influenced transient microbial composition immediately after LF deposition. Among male LFs, 11 of the 32 transient taxa differed between dominant and non-dominant hands under pre-washed conditions, compared with seven taxa following hand washing. For the female, nine of the 28 transient taxa differed between hands before washing, and eleven after washing. Although these differences did not reveal a consistent handedness-specific microbial profile, they suggested that transient taxa were substantially more responsive to the environment than the stable core microbiota. Temporal succession was evident across all donor-condition combinations. For instance, on the dominant pre-washed hand of the female, *Acidobacteria* appeared at T192, but *Cytophagia*, *Deltaproteobacteria*, *Epsilonproteobacteria*, *Fusobacteriia*, *and Negativicutes* were no longer detected. Donor-specific differences in microbial succession were also observed. Overall, the male showed greater temporal variability in transient microbial composition than the female, whereas the female retained fewer transient taxa throughout the experiment. Due to the inherent limitations of the experiment design, these differences should be interpreted as inter-individual variation rather than evidence of sex-specific microbial succession and should be taken cautiously.

**Figure 5:**
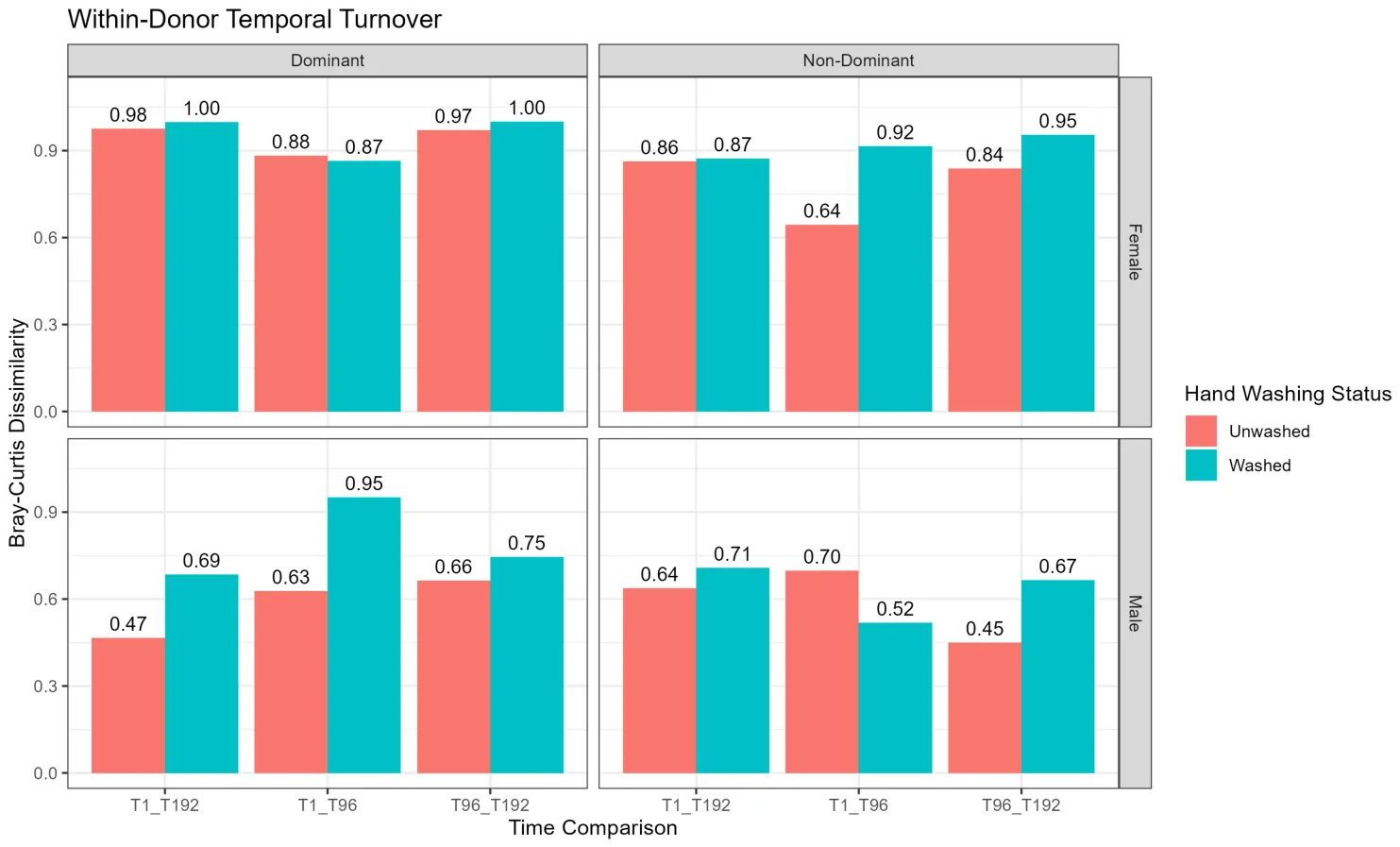
Bray–Curtis dissimilarity charts between time intervals for each donor, handedness, and hand-washing condition. Community turnover was calculated from relative abundance data by comparing T1–T96, T96–T192, and T1–T192. Bray–Curtis dissimilarity ranges from 0 (identical community composition) to 1 (completely dissimilar community composition), with higher values indicating greater temporal turnover of LF-associated microbial communities over time. Data include only the low abundant transient taxa.

Bray–Curtis dissimilarity analyses revealed substantial temporal turnover within all LF microbial communities, with dissimilarity values ranging from 0.45 to 1.00 (Figure 5) and varied among donors, handedness, and hand-washing status. The female showed more dissimilarity across all treatments in comparison to the male, except for the unwashed non-dominant hand, where the male was 6% more dissimilar than the female non-dominant unwashed hand between the first (T1) and second (T96) period, and in the dominant washed hand, where the male showed 8% more dissimilarity than female in that same period. When comparing dissimilarity between handedness, female dominant microbial communities were generally more dissimilar, except for post-washing between the first (T1) and second (T96) period, where LFs from the non-dominant hand were 5% more dissimilar in comparison to the dominant hand. The male handedness presented a more complex story, where LF microbial communities in the non-dominant hand were more dissimilar between the first (T1) and third (T192) period. Male LF communities originating from the dominant hand conversely were more dissimilar between the last two sampling times (T96 and T192). However, the male LF microbial community was 5% more dissimilar in non-dominant than dominant washed hands and 43% more dissimilar in dominant than non-dominant unwashed hands between these time periods. When comparing dissimilarity between washed and unwashed hands, female washed hands were generally more dissimilar than unwashed hands, except for dominant hand LF microbial communities between the first two periods (T1–T96); however, there was only 1% dissimilarity between washing conditions. Male donor washed and unwashed comparisons followed a similar trend, where hand washing generally increased dissimilarity between communities, except for the non-dominant hand between the first two time points (T1 and T96), where non-dominant unwashed hands were 18% more dissimilar than washed non-dominant LF microbial communities at that time point. Despite substantial temporal turnover, microbial communities remained highly differentiated between donors over time, where hand washing generally created more similar communities, except for collection at T96, where hand washing generated, between donors, dissimilarities 6% greater than unwashed communities (Supplemental Figure S5).

**Figure 6:**
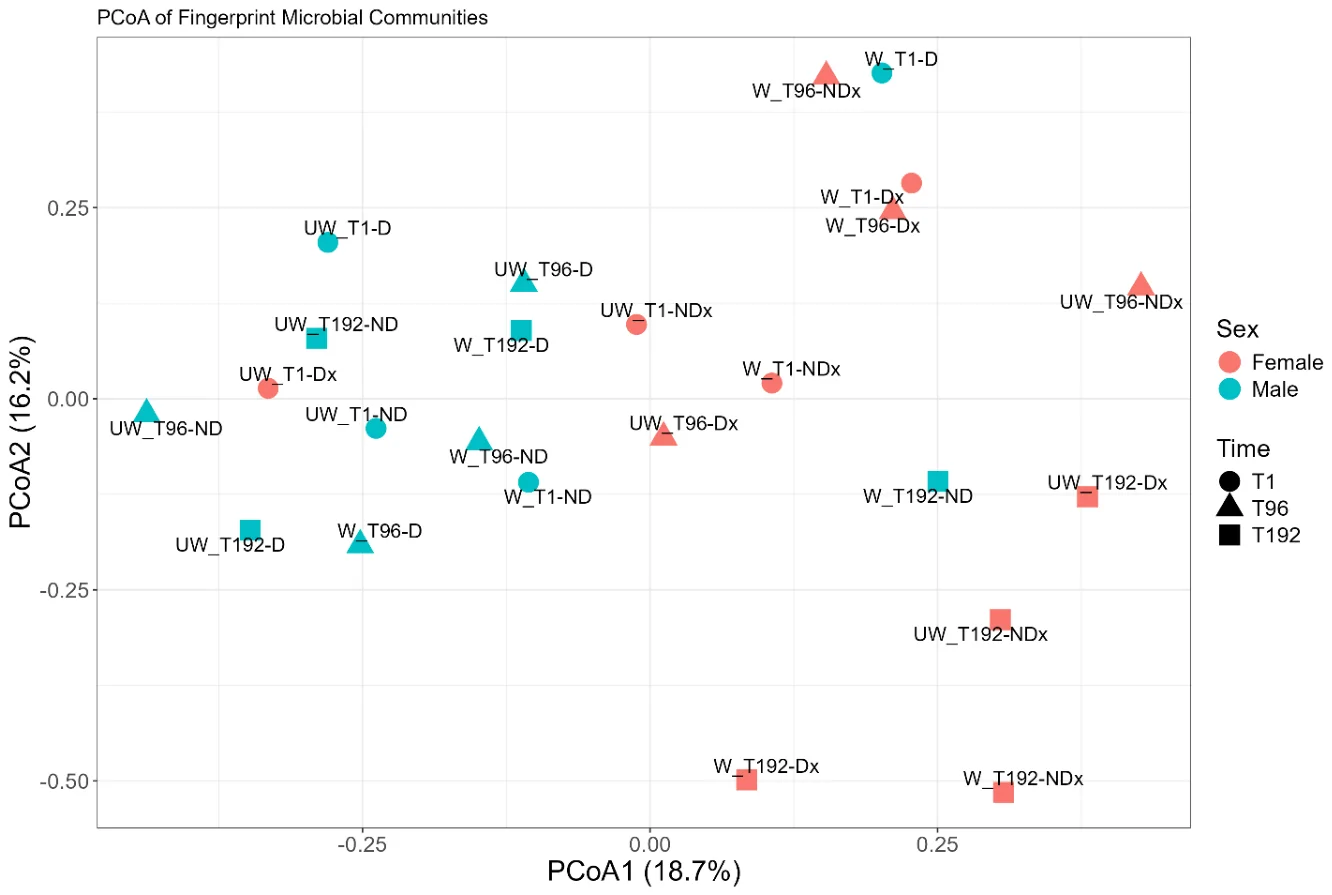
Principal Coordinate Analysis (PCoA) of LF-associated microbial communities based on Bray–Curtis dissimilarities calculated from relative taxonomic abundances. Each point represents an individual LF sample set collected from different donors, time post-deposition, handedness (D = dominant; ND = non-dominant), and hand-washing (W = washed; UW = unwashed) conditions. The distance between points reflects differences in microbial community composition, with samples clustering according to compositional similarity. The first two principal coordinate axes explained 34.9% of the total variation in community structure (PCoA1 = 18.7%; PCoA2 = 16.2%). Colors represent donor identity, and symbols denote post-deposition interval. Data include only the low abundant transient taxa.

Principal Coordinates Analysis (PCoA) based on Bray–Curtis dissimilarities further illustrated there was no distinct clustering across handedness, hand-washing status, or post-deposition interval. Instead, donors represented the primary source of variation along the first principal coordinate, which explained 18.7% of the total community variation, while the second axis accounted for 16.2%, yielding a cumulative explanation of 34.9%, with the exception of one datapoint for the female and two for the male. (Figure 6). Generally, there was some clustering by time, albeit more prevalent in the female donor, suggesting a more robust dataset could further determine if there are distinguished communities by time and donor. Hand-washed samples also occupied a broader ordination space than pre-washed LFs, indicating greater variability following hand washing.

**Figure 7:**
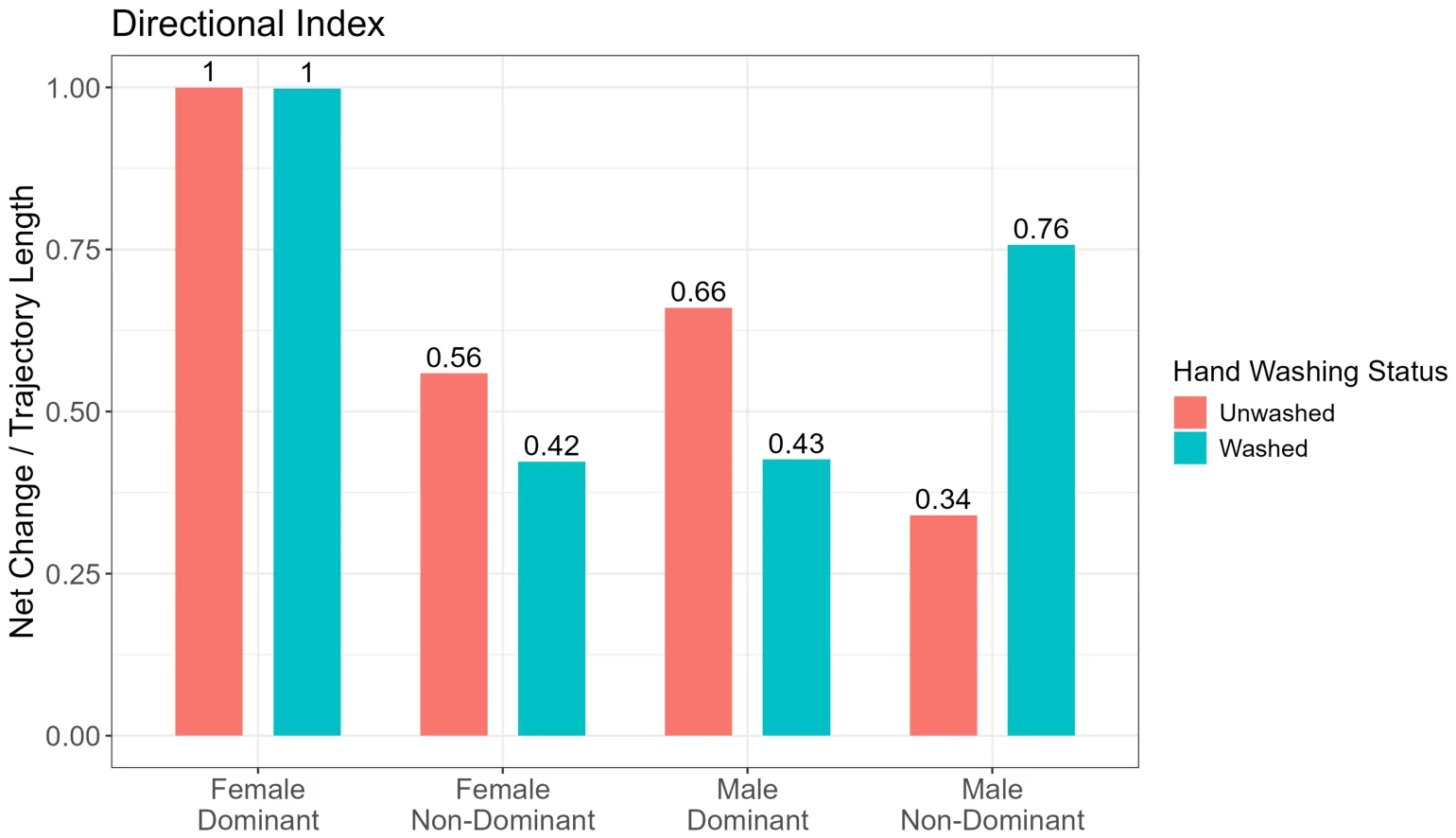
Directional index (DI) chart describing the directionality of LF microbial succession for each donor, handedness, and hand-washing condition. DI was calculated as the ratio of net compositional change to cumulative trajectory length (Net Change/Trajectory Length) from Bray–Curtis-based PCoA trajectories. Values approaching 1 indicate consistent, directional community succession, whereas lower values reflect more complex or non-linear community trajectories. Only transient (time-variant) taxa were included in the analysis following exclusion of the stable core microbiota.

Temporal trajectories reconstructed from Bray–Curtis-based PCoA coordinates revealed considerable differences in both the magnitude and direction of LF microbial succession among donor–condition combinations (Figure 6). These trajectories were quantified with the Directional Index (DI) (Figure 7, Supplemental Table S6). Female dominant-hand communities showed the highest DI values under both washed and unwashed hand conditions (0.999 and 0.998, respectively), indicating highly identical trajectory lengths and net changes. In contrast, female non-dominant communities displayed lower DI values, particularly under washed conditions (0.42), despite having the greatest overall community movement observed in the study. Male LF communities generally showed lower DI values overall, with the lowest value observed for the unwashed non-dominant hand (0.34), reflecting comparatively limited directional change. Hand washing altered these patterns, particularly in male LFs, where the DI increased in the non-dominant hand (0.76) but decreased in the dominant hand (0.43). These results indicated that microbial succession did not follow a single trajectory across donors and conditions but instead proceeded through multiple successional pathways that varied in both magnitude and direction.

Several transient taxa were observed exclusively in one donor (Figure 4, Supplemental Table S5). Female-associated taxa included *Dehalococcoidia*, *Nitrospira*, *Armatimonadetes* (class NA), and *Planctomycetes* (class NA), whereas *Chlamydiae*, *Gracilibacteria*, *Nitriliruptoria*, and *Verrucomicrobiae* were detected only in the male. Likewise, several taxa exhibited temporal associations. Fresh LFs (T1) were characterized by *Dehalococcoidia*, *Nitrospira*, *Spirochaetes*, and *Epsilonproteobacteria*, intermediate-aged LFs (T96) by *Thermoleophilia*, *Sphingobacteriia*, *Acidimicrobiia*, and *Proteobacteria*, and late-stage LFs (T192) by *Acidobacteria*, *Parcubacteria*, *Deltaproteobacteria*, *Planctomycetia*, and *Phycisphaerae* (Figure 4, Supplemental Table S7).

Collectively, these results revealed that, unlike the relatively stable core microbiota, transient LF-associated microbial communities underwent substantial temporal succession, influenced by donor, hand washing, and post-deposition interval. These dynamic microbial populations therefore represent potential candidates for developing microbiome-based models for estimating TsDp. However, due to the limited donor pool, these data should be cautiously interpreted and used to support new research rather than make exclusive determinations regarding temporal taxa.

## 4. Discussion

This proof-of-concept study aimed to develop time-sensitive microbial panels by examining how three key factors: handedness, hand-washing status, and time post-deposition, influenced the composition and temporal discriminatory power of LF-associated microbiota based on a two-donor scenario. LFs were aged in a monitored but uncontrolled dark indoor environment, representing conditions commonly encountered at crime scenes in which LF evidence may be deposited and subsequently recovered. Under this scenario, microbial panels could provide complementary forensic intelligence by assisting in donor differentiation, reconstructing sequences of contact, and estimating when objects were handled. Although 2D and 3D morphometric analyses showed limited temporal sensitivity over the eight-day aging period, microbial community analyses revealed measurable ecological changes, particularly among transient taxa, supporting the hypothesis that the LF chronomicrobiome may represent a promising complementary biological approach for TsDp estimation.

The 2D and 3D morphometric analyses of friction ridges were initially performed to determine whether physical changes in topography could provide sufficient temporal information. Although 2D-BG showed better discriminatory capability than 3D-Sa, neither metric consistently differentiated handedness, hand-washing status, or post-deposition interval across experimental conditions. These preliminary findings indicated that friction ridge morphology remained relatively stable over 8 days, with only subtle changes detectable with the tested morphometrics. Therefore, any temporal variations observed in microbial communities were unlikely to significantly influence any alterations in ridge topography. Rather, the results suggested that biological processes occurring within the deposited microbial community were more dynamic than the physical degradation of the LFs, supporting microbial profiling as a potentially more temporally sensitive approach than 2D and 3D morphometric analyses for estimating TsDp over short time intervals. Recent advances in forensic microbiome research have suggested that the LF chronomicrobiome [29] provides considerable promise as a complementary source of temporal information for addressing TsDp. There is the need for biomarkers capable of differentiating contributors under realistic forensic scenarios involving multiple individuals depositing biological material on the same item of evidence. Such situations commonly arise with handled objects such as knives, firearms, tools, or mobile phones, where reconstructing the chronological sequence of contact may be critical for case interpretation. While morphometric characterization may provide information related to ridge structure and physical degradation, microbial communities are highly dynamic biological systems that respond much quicker to internal and external factors after LF deposition.

Analysis of the LF microbial communities indicated that a few dominant LF-associated taxa remained remarkably stable throughout the study, consistent with previous investigations showing that core skin microbiota displayed substantial temporal stability despite variations in relative abundance resulting from physiological and environmental influences [14,31,34,35,39,41]. While this stability supported the robustness of donor-specific microbial panels, it also suggested that dominant taxa alone possessed limited discriminatory value for estimating TsDp. Previous forensic microbiome studies have largely focused on the taxonomic occurrence and relative abundance of these dominant microorganisms, often overlooking the transient microbial taxa that comprise a smaller but considerably more dynamic component of the community [17,33]. For this reason, the present study focused on transient low-abundant microbes as potential indicators of temporal change following LF deposition. Unlike dominant taxa, transient microorganisms experienced larger variability in their occurrence, composition, and succession through time and may provide increased discriminatory power. The Shannon diversity index revealed dynamic changes in microbial community composition associated with both hand-washing status and elapsed time, even after accounting for the relatively stable core microbiota. These observations suggested that LF microbial communities continue to change after deposition through ecological processes influenced by nutrient availability from LF secretions, moderate indoor environmental conditions, and environmental (microbial) inputs (i.e., eDNA). The identification of transient taxa showing predictable temporal dynamics may provide substantially greater forensic value than dominant taxa because of their quicker response to the environment, which could collectively better contribute to estimating TsDp. Such approaches would rely not only on relative abundance but also on microbial occurrence, community composition, succession patterns, and ecological interactions.

Among the evaluated experimental factors, hand washing produced the strongest effect on overall LF microbiota, consistently reducing microbial diversity. Handedness also influenced microbial composition, although its effects were less pronounced and more variable. Following deposition, pre-wash microbial diversity remained relatively stable, with minor decreases over time, whereas post-wash communities displayed larger variability and, in some cases, increased diversity. These results emphasize the strong influence of hygiene practices on transferred skin microbial communities while also demonstrating donor-specific differences in microbial recovery and community restructuring after disturbance.

These findings demonstrated that LF microbial communities are governed primarily by the individual donor microbiome, with time since deposition representing the second most influential factor once donor-specific variation is considered. Although analyses combining both donors suggested only negligible overall effects, stratified analyses consistently revealed donor-dependent microbial succession, indicating that interpersonal variability masks temporal ecological changes. Hand washing and hand dominance further modified these trajectories by altering microbial persistence and recolonization, particularly when analyzed together with time. The strongest signals were observed within specific combinations of donor, hand-washing status, and handedness, where moderate to large effect sizes demonstrated reproducible temporal changes in microbial community composition. These results support the concept that microbial succession may have considerable potential for estimating TsDp, especially in distinguishing donor profiles, but also indicate that future predictive models should incorporate donor-specific baseline microbiomes and relevant behavioral factors, such as handedness and hygiene, to maximize accuracy and forensic applicability. These findings suggest that the effects of time, handedness, and hand-washing were not uniform between donors but instead reflected donor-specific response patterns.

Community-level ecological analyses further demonstrated that LF microbiomes undergo structured ecological succession following deposition. Bray–Curtis dissimilarity analyses revealed that microbial community turnover differed among donors, handedness, and handwashing conditions, although no single temporal pattern explained all comparisons. Overall, handwashing led to larger temporal community turnover than unwashed conditions, indicating that handwashing substantially altered subsequent successional trajectories. Despite these temporal changes, microbial communities remained clearly differentiated between donors throughout the aging period, demonstrating that the initial donor LF microbiome composition influenced microbial panels.

Unlike previous LF microbiome studies, which have primarily emphasized donor identification based on microbial composition [14,16,18], the present work evaluated microbial succession using ecological community metrics, including Bray–Curtis dissimilarity, community turnover, and trajectory analyses. These approaches demonstrated that microbial succession occurred every time following deposition, although the magnitude and direction of change varied according to donor identity, hand dominance, and hand-washing status. Rather than converging toward a common microbial endpoint, individual communities followed distinct ecological trajectories, indicating that succession was donor- and condition-specific rather than “universal”. Exploratory analyses of candidate taxa further suggested that certain microbial groups were associated with specific donors and aging intervals, supporting the conclusion that LF microbiomes undergo structured but heterogeneous ecological succession, also supporting the need for research involving larger donor pools and mixed environmental conditions to further explore these preliminary results.

The marked influence of hand washing can be interpreted within the framework of ecological disturbance theory. This factor represents a disturbance that removes dominant members of the resident skin microbiota, thereby fundamentally altering the microbial inoculum transferred to the substrate [50]. The immediate reduction or disappearance of previously abundant taxa following washing likely reduced competitive dominance within the community, allowing less abundant microorganisms to contribute more substantially to subsequent succession [51,52]. This hypothesis is supported by the lower between-donor dissimilarity observed immediately after washing, suggesting that donor-specific taxa were initially diminished before microbial communities progressively diverged through time (Supplemental Figure S5).

The observed temporal changes are likewise consistent and may be attributable to classical ecological succession; whereby biological communities develop predictably following a disturbance event [53]. Similar successional processes have been documented in other forensic microbiome applications, including decomposition [42] and soil microbial communities [54], where predictable community turnover provides valuable temporal information. The LF environment constitutes a unique ecological habitat in which microorganisms interact with nutrients derived from eccrine and sebaceous secretions, including amino acids, proteins, lipids, [55] and salts [56], whose composition varies among individuals. Consequently, each deposited LF establishes a distinct microbial microenvironment that influences subsequent community assemblage. Despite evidence of succession, donor-specific microbial communities remained distinguishable throughout the entire aging period. This persistence may indicate that the initial microbial assemblage influences the establishment, persistence, and exclusion of later colonizing taxa through ecological priority effects [57], whereby early colonizers shape subsequent community development. Priority effects have been recognized as major ecological drivers in numerous microbial systems, including soil and plant/animal decomposition microbiomes [58], and likely contribute to the donor-specific community trajectories observed in LFs. However, future research should investigate the priority effects on LFs before making this assertion. Importantly, because *16S rRNA* amplicon sequencing does not distinguish viable microorganisms from non-viable cells or extracellular DNA, the observed successional patterns likely reflect temporal changes in the overall microbial panel rather than active microbial growth alone. These profiles probably comprise combinations of resident skin microbiota, environmentally deposited microorganisms (eDNA), and extracellular DNA [59]. Nevertheless, the persistence of donor-specific community structure together with distinct ecological trajectories indicates that LF microbial panels tend to change in an organized, non-random manner through time.

To illustrate the potential forensic application of these findings, a hypothetical case scenario was constructed with combinations of transient microbial indicator taxa. Although intended solely as a purely conceptual illustration with no forensic decision-making value, the example depicts how “microbial panels” could progressively exclude potential contributors and deposition time intervals. For instance, a microbial profile consisting of *Acidimicrobiia (−)*, *Acidobacteria (+)*, *Actinobacteria (−)*, *Anaerolineae (−)*, *Chlamydiae (+)*, *Chlorobia (−)*, *Chloroflexi* (NA), *Coriobacteriia (−)*, *Cytophagia (−)*, *Deinococci (−)*, *Deltaproteobacteria* (+), and *Epsilonproteobacteria* (−) immediately excluded one donor because this combination was not observed under any of that individual’s experimental conditions. For the remaining donor, the same microbial panel eliminated multiple deposition intervals, ultimately identifying the 192 h sample as the closest matching profile. Although this example is illustrative rather than predictive, it demonstrates how combinations of transient microbial taxa may eventually support probabilistic reconstruction of donor identity and approximate deposition time within realistic forensic investigations.

The donor-specific microbial patterns observed in this study are also consistent with previous reports describing sex-related differences in skin microbiota stability, which have been attributed to differences in sebum production, hormonal regulation, sweat composition, hygiene practices, and cosmetic use [15,60,61,62,63]. However, because the present experiment included only two donors, these observations should be interpreted cautiously and cannot be generalized without validation in substantially larger and more diverse study populations.

The data show that donor identity establishes the baseline microbial composition, reflecting each individual’s “unique” skin microbiome. Time since deposition then drives ecological succession within that donor-specific community through differential microbial survival and adaptation to the new LF environment. Hand washing modifies this succession by removing transient microorganisms and altering the initial biological and chemical conditions of the LF, thereby influencing the subsequent recolonization process. Finally, handedness contributes comparatively little additional variation, as both hands share the same resident microbiota and differ mainly in environmental exposure and contact frequency. These findings support the concept that any potentially accurate estimation of TsDp requires accounting for the strong donor-specific microbial baseline and the modifying effects of hygiene-related behaviors.

Overall, this study provides a forensic and ecological framework for understanding LF-associated microbial succession under realistic indoor conditions. While friction ridge morphology remained comparatively stable and showed less temporal discriminatory capability over the eight-day aging period, microbial communities experienced structured temporal succession driven primarily by transient taxa, with donor identity, hand-washing status, and elapsed time since deposition emerging as the principal factors of microbial community dynamics. These preliminary data establish the LF *chronomicrobiome* as a promising complementary source of forensic intelligence for estimating TsDp and reconstructing contact events.

The present proof-of-concept study was intentionally designed to replicate a realistic forensic scenario involving a limited number of contributors rather than to investigate population-level microbial variability. The main objective was to assess the feasibility of employing LF microbial succession as a temporal indicator of TsDp rather than to develop a generalized predictive model. Therefore, these results should be regarded as preliminary and hypothesis-generating, providing a basis for future studies incorporating larger donor cohorts, additional substrates, a broader range of environmental conditions, a distinction between viable microorganisms from extracellular DNA, longer aging periods, and the development of machine-learning models capable of translating microbial succession into robust, probabilistic estimates of LF age and TsDp. Likewise, the proposed candidate taxa should be regarded as preliminary biomarkers requiring further validation in future investigations rather than verifying certain taxa unequivocally associated with specific donors or deposition intervals.

## 5. Conclusions

These preliminary findings support the hypothesis that microbial succession within LFs follows structured ecological, but heterogenous, succession, which ultimately may be the basis for a forensic “biological clock” in the future. While dominant microbial taxa remained comparatively stable throughout the eight-day aging period, transient taxa showed considerably greater temporal variability, referred to as “microbial panels”. This variability was influenced primarily by donor-specific microbial identity, elapsed time since deposition, and hand washing, whereas handedness exerted comparatively smaller effects. Future research should prioritize investigating microbial viability, analyzing longer elapsed times, discerning environmental DNA from skin-transferred DNA, and developing robust predictive models for time-since-deposition (TsDp) estimation.

## Figures and Tables

**Table 1:** Summary of Kruskal-Wallis tests (*p*-values) for the selected microbial signatures at the class taxonomic rank per donor and both combined. Alpha = 0.05. The table is organized by splitting each data set into one, two, and three factors. T1 = 3–6 h; T96 = 96 h; T192 = 192 h post-deposition. Bold and highlighted values indicate statistical significance. (*) represents marginally significant values (n = 780). (§) represent moderate or large effects (η^2^H ≥ 0.06).

			Combined Donors					Male					Female				
Factor Combination			Abundance		Occurrence		n	Abundance		Occurrence		n	Abundance		Occurrence		n
1st Factor	2nd Factor	3rd Factor	*p*-Value	η^2^H	*p*-Value	η^2^H		*p*-Value	η^2^H	*p*-Value	η^2^H		*p*-Value	η^2^H	*p*-Value	η^2^H	
Handedness	- - - - -	- - - - -	0.116	0.002	0.250	0.000	780	0.243	0.001	0.280	0.000	390	0.265	0.001	0.602	0.000	390
Hand Washing	- - - - -	- - - - -	0.149	0.001	0.107	0.002	780	0.197	0.002	0.092	0.005	390	0.729	0.000	0.601	0.000	390
Time	- - - - -	- - - - -	0.285	0.001	0.158	0.002	780	**0.007**	0.020	**0.016**	0.020	390	**0.004**	0.024	**<0.001**	0.034	390
Handedness	Pre-washed	- - - - -	0.865	0.000	0.904	0.000	336	0.250	0.002	0.202	0.004	168	0.097	0.011	0.175	0.005	168
	Post-Washed	- - - - -	0.051 *	0.006	0.092	0.004	444	**0.014**	0.023	**0.008**	0.028	222	0.935	0.000	0.598	0.000	222
	T1	- - - - -	0.449	0.000	0.487	0.000	260	0.685	0.000	0.841	0.000	130	0.487	0.000	0.442	0.000	130
	T96	- - - - -	0.139	0.005	0.167	0.004	260	0.107	0.013	0.156	0.008	130	0.539	0.000	0.613	0.000	130
	T192	- - - - -	0.052 *	0.011	0.173	0.003	260	0.435	0.000	0.567	0.000	130	**0.028**	0.030	0.110	0.012	130
Handedness	Pre-washed	T1	0.438	0.000	0.427	0.000	112	0.629	0.000	0.580	0.000	56	0.522	0.000	0.571	0.000	56
		T96	0.492	0.000	0.405	0.000	112	0.072	0.041	0.060	0.047	56	0.224	0.009	0.279	0.003	56
		T192	0.889	0.000	0.821	0.000	112	0.547	0.000	0.401	0.000	56	0.242	0.007	0.392	0.000	56
	Post-Washed	T1	0.062	0.017	0.077	0.015	148	0.293	0.002	0.363	0.000	74	0.093	0.025	0.119	0.020	74
		T96	**0.014**	0.035	**0.010**	0.039	148	**0.002**	**0.124** ^§^	**<0.001**	**0.157** ^§^	74	0.720	0.000	0.724	0.000	74
		T192	**0.015**	0.034	**0.042**	0.022	148	0.130	0.018	0.119	0.020	74	0.055 *	0.037	0.177	0.012	74
Hand Washing	Dominant	- - - - -	0.805	0.000	0.759	0.000	390	0.420	0.000	0.488	0.000	195	0.244	0.002	0.186	0.004	195
	Non-dominant	- - - - -	0.066	0.006	**0.044**	0.008	390	**0.007**	0.033	**0.002**	0.047	195	0.473	0.000	0.531	0.000	195
	T1	- - - - -	**0.017**	0.018	**0.037**	0.013	260	**0.014**	0.040	**0.016**	0.035	130	0.318	0.000	0.527	0.000	130
	T96	- - - - -	0.953	0.000	0.783	0.000	260	0.485	0.000	0.925	0.000	130	0.587	0.000	0.524	0.000	130
	T192	- - - - -	0.858	0.000	0.689	0.000	260	0.483	0.000	0.397	0.000	130	0.395	0.000	0.632	0.000	130
Hand Washing	Dominant	T1	**0.002**	0.068	**0.006**	0.051	130	**0.011**	**0.086** ^§^	**0.018**	**0.073** ^§^	65	0.067	0.037	0.130	0.021	65
		T96	0.152	0.008	0.164	0.007	130	**0.006**	**0.106** ^§^	**0.008**	**0.097** ^§^	65	0.210	0.009	0.244	0.006	65
		T192	0.412	0.000	0.465	0.000	130	0.649	0.000	0.578	0.000	65	0.404	0.000	0.624	0.000	65
	Non-dominant	T1	0.679	0.000	0.807	0.000	130	0.338	0.000	0.343	0.000	65	0.789	0.000	0.578	0.000	65
		T96	0.103	0.013	0.054 *	0.021	130	**0.033**	**0.056** ^§^	**0.008**	**0.095** ^§^	65	0.574	0.000	0.736	0.000	65
		T192	0.212	0.004	0.153	0.008	130	0.131	0.020	0.071	0.036	65	0.692	0.000	0.886	0.000	65
Time	Pre-washed	- - - - -	0.232	0.003	0.156	0.005	336	0.735	0.000	0.719	0.000	168	**0.034**	0.029	**0.012**	0.041	168
	Post-Washed	- - - - -	0.321	0.000	0.369	0.000	444	**0.002**	0.046	**0.005**	0.040	222	0.076	0.014	**0.032**	0.022	222
	Dominant	- - - - -	0.332	0.000	0.389	0.000	390	**0.006**	0.043	**0.020**	0.031	195	0.340	0.001	0.247	0.004	195
	Non-dominant	- - - - -	0.111	0.006	0.091	0.007	390	0.453	0.000	0.469	0.000	195	**0.001**	**0.059** ^§^	**<0.001**	**0.069** ^§^	195
Time	Dominant	Pre-washed	0.240	0.005	0.140	0.012	168	0.718	0.000	0.615	0.000	84	0.256	0.009	0.163	0.020	84
		Post-Washed	**0.008**	0.035	**0.021**	0.026	222	**<0.001**	**0.170** ^§^	**<0.001**	**0.155** ^§^	111	0.297	0.004	0.554	0.000	111
	Non-dominant	Pre-washed	0.517	0.000	0.544	0.000	168	0.313	0.004	0.258	0.009	84	0.111	0.029	0.062	0.044	84
		Post-Washed	0.114	0.011	0.065	0.016	222	0.901	0.000	0.847	0.000	111	**0.013**	**0.062** ^§^	**0.008**	**0.072** ^§^	111

## Data Availability

The datasets generated and analyzed during this study, including microbial community profiling data and associated metadata, are available from the corresponding author upon reasonable request. Requests for access to the data should be directed to the corresponding author.

## Supplementary Materials

The following supporting information can be downloaded at: https://www.mdpi.com/article/10.3390/genes17080931/s1. Figure S1: Boxplot depicting normalized 3D-Sa values (i.e., average ridge height) by hand-washing condition, handedness, and donor over time. Figure S2: Boxplot depicting 2D-BG values (i.e., ridge clarity) by hand-washing condition, handedness, and donor over time. Figure S3: Boxplot depicting relative abundances (log) of the most ubiquitous LF microbial taxa by handedness and time-post deposition before hand washing. Figure S4: Boxplot depicting relative abundances (log) of the most ubiquitous LF microbial taxa by handedness and time post-deposition after hand washing. Figure S5: Bray–Curtis dissimilarity between donor microbial communities at matched aging intervals, handedness categories, and hand-washing conditions. Table S1: Summary of Kruskal–Wallis tests (*p*-values) for 3D-Sa and 2D-BG metrics when combining both donors (male and female). Table S2: Summary of Kruskal–Wallis tests (*p*-values) for the 3D-Sa metric per donor. Table S3: Summary of Kruskal–Wallis tests (*p*-values) for 2D-BG metric per donor. Table S4: Relative abundance (% Ab.) of the seven most common and time-invariant taxonomic classes by handedness, hand-washing condition, donor, and time post-deposition. Table S5: Summary of Kruskal–Wallis tests (*p*-values) for the entire microbial community at the class taxonomic rank per donor and combined. Table S6: Exploratory donor-associated candidate taxa identified from mean relative abundance patterns. Table S7: Exploratory time-associated candidate taxa identified from mean relative abundance patterns across T1, T96, and T192 samples.

## Abbreviations

The following abbreviations are used in this manuscript:

OP	Optical profilometer
2D-BG	Two-dimensional blue green morphometric
3D-Sa	Three-dimensional average friction ridge height
LF	Latent fingerprint (fingermark)
TsDp	Time since deposition
