# Supplementary material for: Temporal Dynamics of Latent Fingerprint Microbiomes: A First Step to Decoding Crime Evidence
Source: Genes (Basel). 2026 Aug 10;17(8):931. doi: 10.3390/genes17080931 (PMC13512604; doi:10.3390/genes17080931)
Supplement: Supplementary file 1 [file genes-17-00931-s001.zip › genes-4477657-supplementary.pdf]

Supplementary Materials

Figure S1. Boxplot depicting normalized *3D-Sa* values (i.e., average ridge height) by hand washing condition, handedness, and donor over time. Circles denote non-extreme outliers as defined by the statistical software IBM®SPSS® (1.5× interquartile range). Each box includes data from 48 LFs, except for T192, which includes 24 samples. (n = 384)

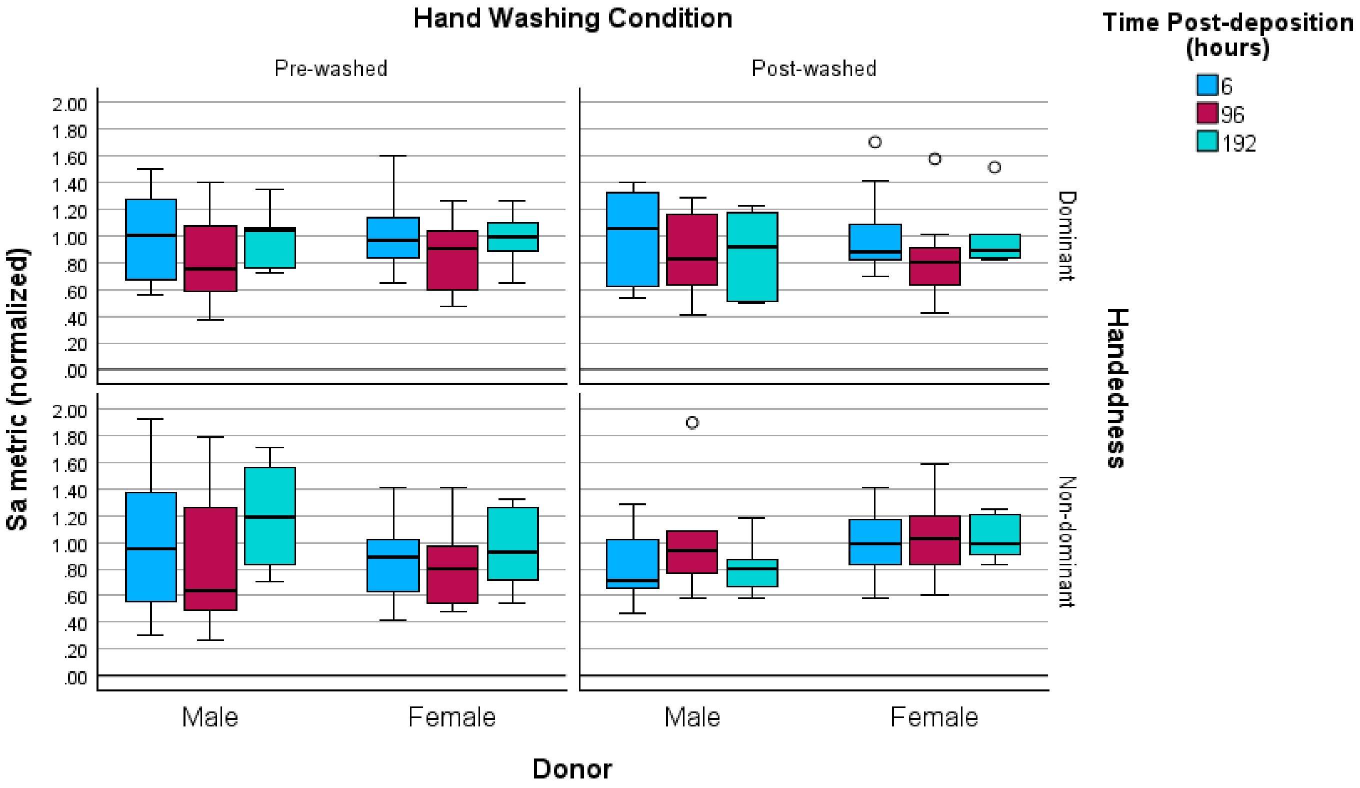

Figure S2. Boxplot depicting *2D-BG* values (i.e., ridge clarity) by hand washing condition, handedness, and donor over time. Circles denote non-extreme outliers as defined by the statistical software IBM® SPSS® (1.5× interquartile range); the asterisks denote 3× interquartile range. Each box includes data from 24 LFs. (n = 348)

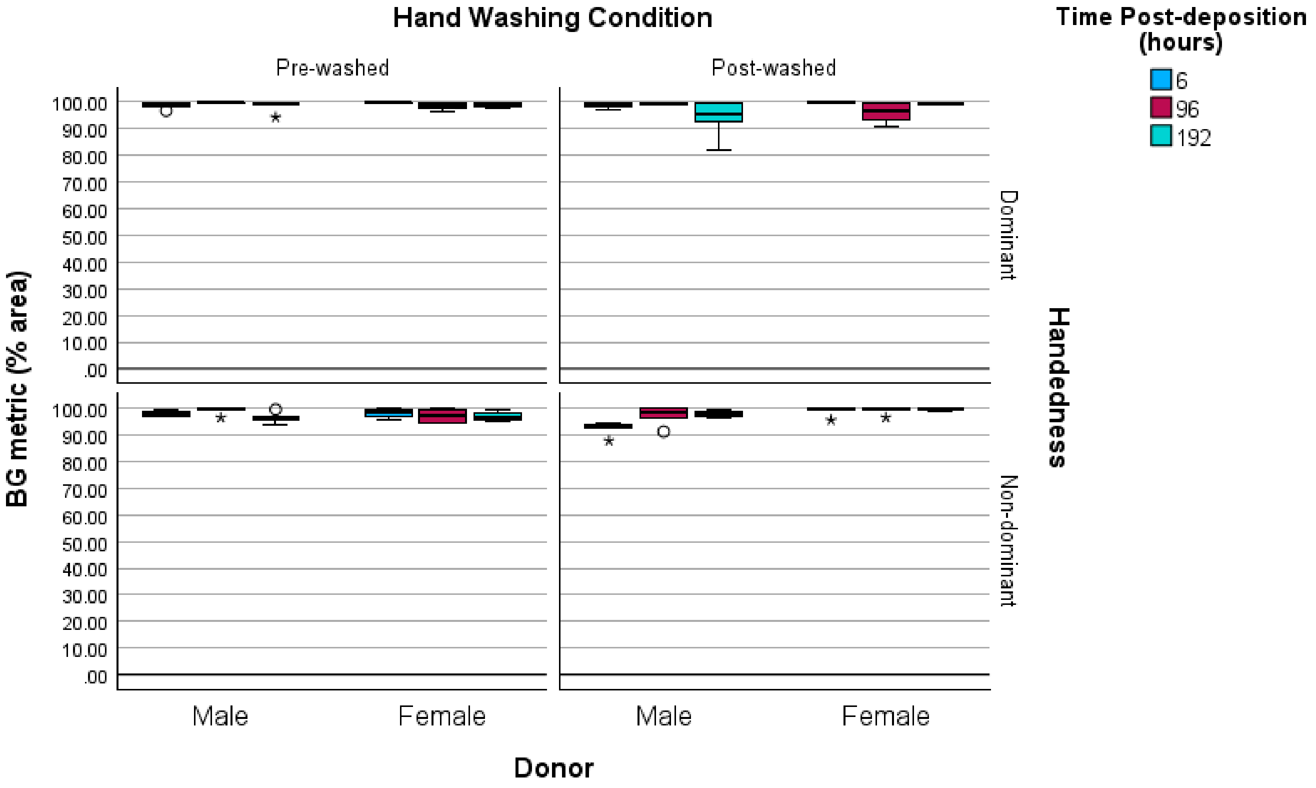

Table S1. Summary of Kruskal-Wallis tests (p-values) for *3D-Sa* and *2D-BG* metrics when combining both donors (male and female). Alpha = 0.05. The table is organized by splitting each data set into one, two, and three combined factors. T1 = 6h; T96 = 96h; T192 = 192h post-deposition. Bold and highlighted values indicate statistical significance.

Factor Combination			*3D-Sa* metric		*2D-BG* metric	
1st Factor	2nd Factor	3rd Factor	p-value	N	p-value	N
**Donor**	- - - - -	- - - - -	0.489	237	**0.006**	141
**Handedness**	- - - - -	- - - - -	0.644	240	**0.003**	144
**Hand Washing**	- - - - -	- - - - -	0.551	240	0.452	144
**Time**	- - - - -	- - - - -	0.074	237	0.290	141
**Handedness**	**Pre-washed**	- - - - -	0.743	120	**<0.001**	72
	**Post-Washed**	- - - - -	0.308	120	0.467	72
	**T1**	- - - - -	0.464	96	**0.001**	48
	**T96**	- - - - -	0.200	96	0.813	48
	**T192**	- - - - -	0.821	48	0.110	48
**Handedness**	**Pre-washed**	**T1**	0.606	48	**0.009**	24
		**T96**	0.837	48	0.184	24
		**T192**	0.686	24	**0.008**	24
	**Post-Washed**	**T1**	0.650	48	0.050	24
		**T96**	**0.041**	48	0.285	24
		**T192**	1.000	24	0.977	24
**Hand Washing**	**Dominant**	- - - - -	0.757	120	0.550	72
	**Non-dominant**	- - - - -	0.283	120	0.131	72
	**T1**	- - - - -	0.930	96	0.853	48
	**T96**	- - - - -	0.099	96	0.765	48
	**T192**	- - - - -	0.201	48	0.228	48
**Hand Washing**	**Dominant**	**T1**	0.951	48	0.795	24
		**T96**	0.967	48	0.371	24
		**T192**	0.488	24	0.751	24
	**Non-dominant**	**T1**	0.869	48	0.273	24
		**T96**	**0.025**	48	0.119	24
		**T192**	0.248	24	**0.009**	24
**Time**	**Pre-washed**	- - - - -	**0.034**	119	0.140	70
	**Post-Washed**	- - - - -	0.753	118	0.826	71
	**Dominant**	- - - - -	**0.023**	120	0.076	72
	**Non-dominant**	- - - - -	0.569	117	0.239	69
**Time**	**Dominant**	**Pre-washed**	0.114	60	0.483	36
		**Post-Washed**	0.182	60	0.124	36
	**Non-dominant**	**Pre-washed**	0.216	59	0.108	34
		**Post-Washed**	0.672	58	0.365	35

Table S2. Summary of Kruskal-Wallis tests (p-values) for the *3D-Sa* metric per donor. Alpha = 0.05. The table is organized by splitting each data set into one, two, and three factors. T1 = 6h; T96 = 96h; T192 = 192h post-deposition. No statistical significance was detected for the cases examined.

***3D-Sa* metric**						
**Factor Combination**			**Male**		**Female**	
**1st Factor**	**2nd Factor**	**3rd Factor**	**p-value**	**N**	**p-value**	**N**
**Handedness**	- - - - -	- - - - -	0.923	118	0.702	119
**Hand Washing**	- - - - -	- - - - -	0.851	118	0.398	119
**Time**	- - - - -	- - - - -	0.404	118	0.162	119
**Handedness**	**Pre-washed**	- - - - -	0.859	60	0.339	59
	**Post-Washed**	- - - - -	0.663	58	0.051	60
	**T1**	- - - - -	0.338	47	0.702	47
	**T96**	- - - - -	0.640	47	0.274	48
	**T192**	- - - - -	0.644	24	0.908	24
**Handedness**	**Pre-washed**	**T1**	0.817	24	0.324	23
		**T96**	0.862	24	0.954	24
		**T192**	0.336	12	0.749	12
	**Post-Washed**	**T1**	0.295	23	0.525	24
		**T96**	0.622	23	0.050	24
		**T192**	1.000	12	0.336	12
**Hand Washing**	**Dominant**	- - - - -	0.988	60	0.280	60
	**Non-dominant**	- - - - -	0.852	58	0.054	59
	**T1**	- - - - -	0.670	47	0.469	47
	**T96**	- - - - -	0.167	47	0.536	48
	**T192**	- - - - -	0.094	24	0.954	24
**Hand Washing**	**Dominant**	**T1**	0.908	24	0.773	24
		**T96**	0.453	24	0.386	24
		**T192**	0.748	12	0.423	12
	**Non-dominant**	**T1**	0.622	23	0.267	23
		**T96**	0.157	23	0.093	24
		**T192**	0.078	12	0.749	12
**Time**	**Pre-washed**	- - - - -	0.084	60	0.407	59
	**Post-Washed**	- - - - -	0.745	60	0.320	60
	**Dominant**	- - - - -	0.173	60	0.113	60
	**Non-dominant**	- - - - -	0.720	58	0.721	59
**Time**	**Dominant**	**Pre-washed**	0.265	30	0.497	30
		**Post-Washed**	0.343	30	0.143	30
	**Non-dominant**	**Pre-washed**	0.331	30	0.720	29
		**Post-Washed**	0.432	28	0.944	30

Table S3. Summary of Kruskal-Wallis tests (p-values) for *2D-BG* metric per donor. Alpha = 0.05. The table is organized by splitting each data set into one, two, and three factors. T1 = 6h; T96 = 96h; T192 = 192h post-deposition. Bold and highlighted values indicate statistical significance.

***2D-BG* metric**						
**Factor combination**			**Male**		**Female**	
**1st Factor**	**2nd Factor**	**3rd Factor**	**p-value**	**N**	**p-value**	**N**
**Handedness**	-----	-----	**0.002**	70	0.441	71
**Hand Washing**	-----	-----	**0.022**	70	**0.002**	71
**Time**	-----	-----	**0.005**	70	**0.031**	71
**Handedness**	**Pre-washed**	-----	**0.012**	35	**0.017**	35
	**Post-Washed**	-----	0.051	35	0.526	36
	**T1**	-----	**0.005**	23	**0.043**	24
	**T96**	-----	0.479	23	0.230	23
	**T192**	-----	0.273	24	0.355	24
**Handedness**	**Pre-washed**	**T1**	0.109	12	0.065	12
		**T96**	0.465	11	0.361	11
		**T192**	0.055	12	0.055	12
	**Post-Washed**	**T1**	**0.006**	11	0.330	12
		**T96**	0.470	12	**0.045**	12
		**T192**	0.522	12	0.810	12
**Hand Washing**	**Dominant**	-----	**0.021**	36	0.201	36
	**Non-dominant**	-----	0.174	34	**0.001**	35
	**T1**	-----	0.079	23	0.14	24
	**T96**	-----	**0.031**	23	0.295	23
	**T192**	-----	0.386	24	**0.007**	24
**Hand Washing**	**Dominant**	**T1**	0.470	12	0.147	12
		**T96**	0.109	12	0.522	12
		**T192**	0.109	12	0.201	12
	**Non-dominant**	**T1**	**0.006**	11	0.336	12
		**T96**	0.465	11	**0.045**	11
		**T192**	0.200	12	**0.010**	12
**Time**	**Pre-washed**	-----	**0.014**	35	0.093	35
	**Post-Washed**	-----	0.093	35	0.193	36
	**Dominant**	-----	0.171	36	**<0.001**	36
	**Non-dominant**	-----	**0.037**	34	0.844	35
**Time**	**Dominant**	**Pre-washed**	0.279	18	**0.035**	18
		**Post-Washed**	0.181	18	**0.019**	18
	**Non-dominant**	**Pre-washed**	**0.048**	17	0.426	17
		**Post-Washed**	**0.023**	17	0.848	18

Figure S3. Boxplot depicting relative abundances (log) of the most ubiquitous LF microbial taxa by handedness and time-post deposition before hand washing. Asterisks denote a 3 × interquartile range as defined by the statistical software IBM® SPSS®. Each box includes data from 6 LFs and is organized by decreasing abundance from left to right. (Each plot represents n = 6)

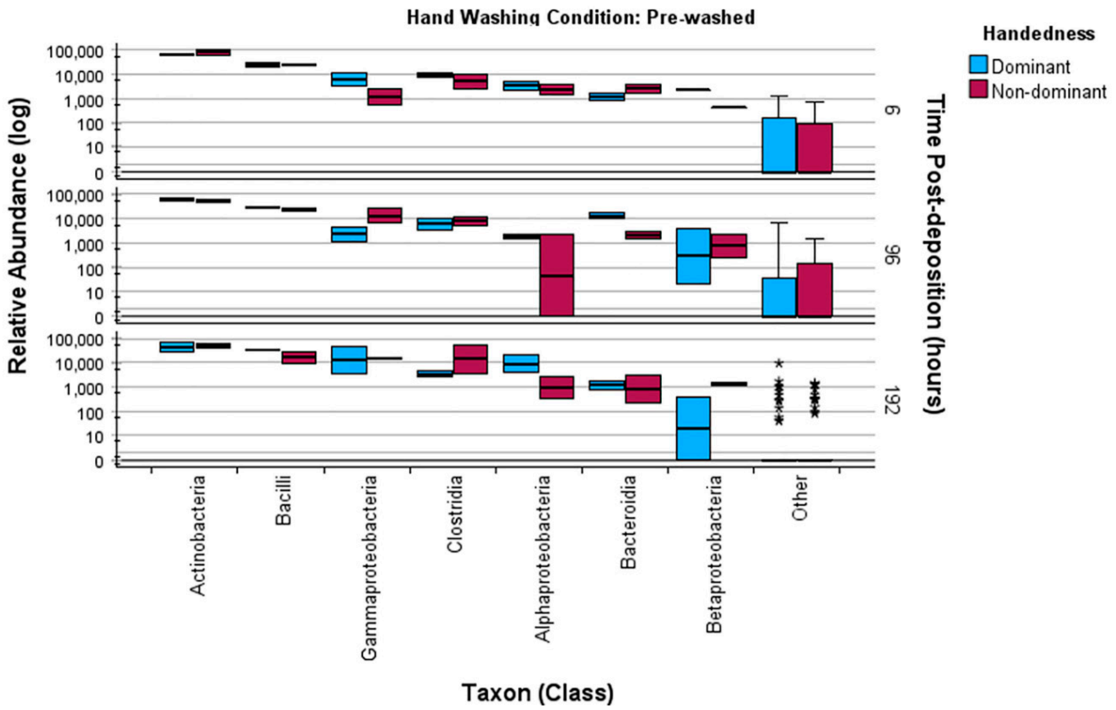

Figure S4. Boxplot depicting relative abundances (log) of the most ubiquitous LF microbial taxa by handedness and time-post deposition after hand washing. Asterisks denote a 3 × interquartile range as defined by the statistical software IBM® SPSS®. Each box includes data from 6 LF and is organized by decreasing abundance from left to right. Each plot represents n = 6)

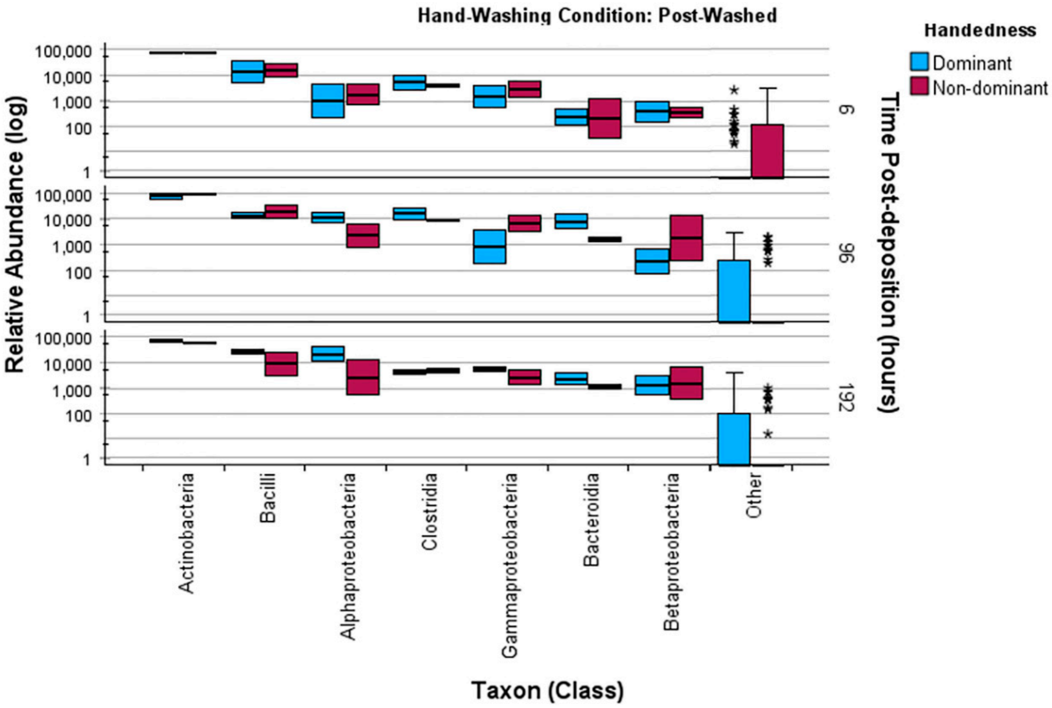

Table S4. Relative abundance (% Ab.) of the seven most common and time-invariant taxonomic classes by handedness, hand washing condition, donor, and time post-deposition. The percentage difference (% Diff.) is between dominant (D) and non-dominant (ND) hands in absolute numbers. T1 = 6h; T96 = 96h; T192 = 192 h post-deposition. The values are the average of two replicates. Highlighted are the lowest abundances of the combined seven invariant major taxa.

	**Male Donor**											
	**Pre-washed Hands**						**Post-washed Hands**					
	**T1**		**T96**		**T192**		**T1**		**T96**		**T192**	
	**D**	**ND**	**D**	**ND**	**D**	**ND**	**D**	**ND**	**D**	**ND**	**D**	**ND**
**% Ab.**	96.70 (± 1.45)	**83.09** (± 20.77)	94.84 (± 0.75)	95.47 (± 0.57)	96.85 (± 1.00)	96.62 (± 0.21)	95.49 (± 2.19)	93.88 (± 5.51)	**84.71** (± 4.86)	97.20 (± 2.31)	94.49 (± 5.28)	98.01 (± 0.69)
**% Diff.**	|13.62|		|0.64|		|0.23|		|1.61|		|12.49|		|3.52|	

	**Female Donor**											
	**Pre-washed Hands**						**Post-washed Hands**					
	**T1**		**T96**		**T192**		**T1**		**T96**		**T192**	
	**D**	**ND**	**D**	**ND**	**D**	**ND**	**D**	**ND**	**D**	**ND**	**D**	**ND**
**% Ab.**	97.66 (± 0.18)	97.31 (± 0.95)	95.98 (± 3.46)	98.60 (± 1.98)	94.23 (± 2.43)	97.71 (± 3.24)	99.69 (± 0.36)	98.41 (± 0.88)	98.59 (± 1.48)	96.26 (± 4.33)	**89.19** (± 14.27)	99.62 (± 0.53)
**% Diff.**	|0.35|		|2.62|		|3.48|		|1.28|		|2.33|		|10.43|	

Table S5. Summary of Kruskal-Wallis tests (p-values) for the entire microbial community at the class taxonomic rank per donor and combined. Alpha = 0.05. The table is organized by splitting each data set into one and three factors. T1 = 3-6h; T96 = 96h; T192 = 192h post-deposition. Bold and highlighted values represent statistical significance. (*) represent marginally significant values.

			Combined Donors		Male		Female	
Factor combination			Abundance	Presence	Abundance	Presence	Abundance	Presence
1st Factor	2nd Factor	3rd Factor	p-value	p-value	p-value	p-value	p-value	p-value
**Handedness**	-----	-----	0.226	0.351	0.402	0.356	0.389	0.694
**Hand Washing**	-----	-----	**0.036**	**0.036**	0.145	**0.042**	0.227	0.357
**Time**	-----	-----	0.548	0.319	**0.055***	**0.050**	0.106	**0.011**
**Handedness**	**Pre-washed**	**T1**	0.523	0.500	0.703	0.635	0.571	0.634
		**T96**	0.787	0.494	0.282	0.096	0.818	0.809
		**T192**	0.994	0.727	0.649	0.476	0.611	0.790
	**Post-Washed**	**T1**	0.150	0.205	0.425	0.491	0.196	0.274
		**T96**	0.076	**0.031**	**0.020**	**0.001**	0.818	0.809
		**T192**	0.063	0.110	0.295	0.192	0.121	0.341
**Hand Washing**	**Dominant**	**T1**	**0.004**	**0.009**	**0.040**	**0.029**	**0.052***	0.132
		**T96**	0.543	0.313	0.076	**0.025**	0.238	0.370
		**T192**	0.721	0.707	0.897	0.819	0.661	0.757
	**Non-dominant**	**T1**	0.393	0.475	0.392	0.296	0.702	0.977
		**T96**	0.170	0.079	0.110	**0.011**	0.960	0.877
		**T192**	0.181	0.133	0.190	0.080	0.718	0.759
**Time**	**Dominant**	**Pre-washed**	0.458	0.194	0.898	0.710	0.443	0.207
		**Post-Washed**	**0.033**	0.067	**0.002**	**<0.001**	0.457	0.722
	**Non-dominant**	**Pre-washed**	0.801	0.733	0.608	0.346	0.429	0.207
		**Post-Washed**	0.422	0.255	0.933	0.967	0.164	0.095

Figure S5. Bray-Curtis dissimilarity between donor microbial communities at matched aging intervals, handedness categories, and hand-washing conditions. Values closer to 1 indicate larger differentiation in community composition between donors at that time. Lines connect measurements across aging intervals to illustrate donor-specific microbial community structure persistence over time.

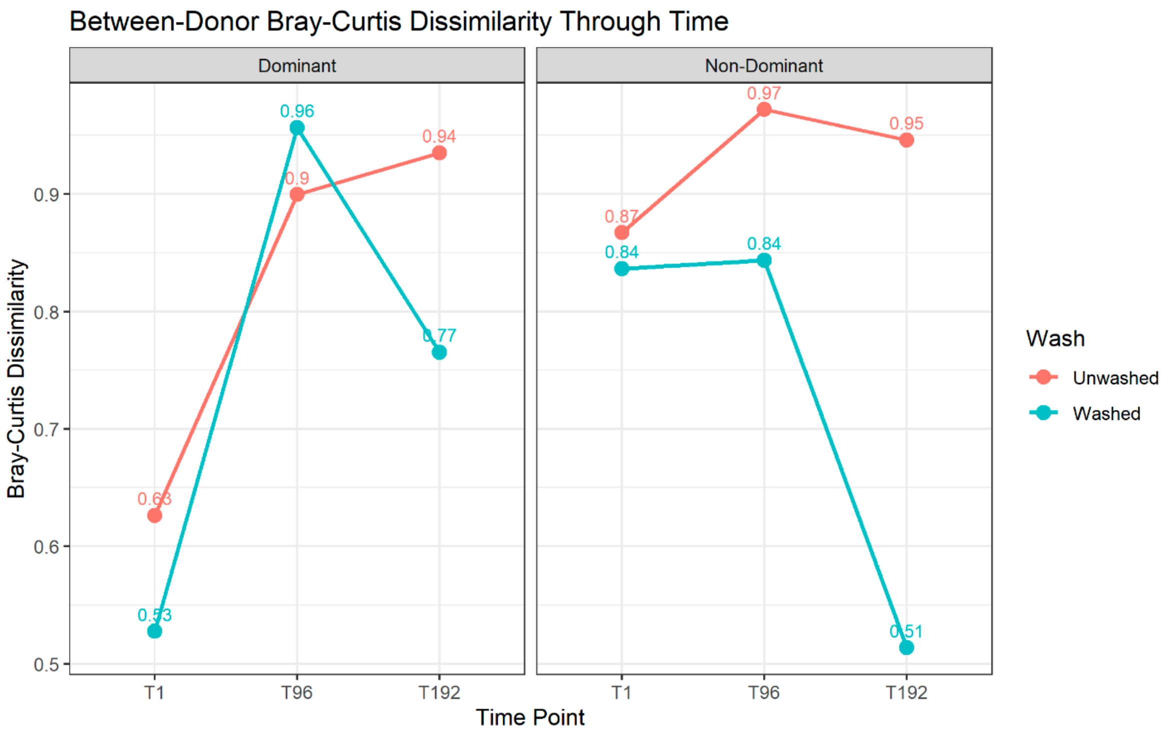

Table S6. Exploratory donor-associated candidate taxa identified from mean relative abundance patterns. Specificity represents the proportion of total abundance attributable to the donor exhibiting the highest mean abundance. Because two donors were included in this study, these taxa should be interpreted as donor-associated candidates rather than validated donor indicators. (*) depicts taxa detected in one donor only.

Taxon	Donor	Max_Abundance	Specificity
Armatimonadetes;c__NA	Female	0.002163	1
Dehalococcoidia	Female	0.018547	1
Nitrospira	Female	0.00296	1
Planctomycetes;c__NA	Female	0.010221	1
Epsilonproteobacteria	Female	0.018083	0.933156
Coriobacteriia	Female	0.13614	0.929147
Planctomycetacia	Female	0.061536	0.864866
Thermoleophilia	Female	0.080029	0.704578
Acidobacteria	Female	0.120419	0.680738
Parcubacteria;NA*	Female	0.11424	0.675993
Thermomicrobia	Female	0.011897	0.671068
Sphingobacteriia	Female	0.12948	0.596443
Deinococci	Female	0.01431	0.573828
Gemmatimonadetes	Female	0.001623	0.537059
Actinobacteria;c__NA	Male	0.00109	1
Chlamydiae	Male	0.002055	1
Chlorobia	Male	0.000931	1
Gracilibacteria*	Male	0.003344	1
Nitriliruptoria	Male	0.001535	1
Proteobacteria*	Male	0.004804	1
Verrucomicrobia*	Male	0.001276	1
Verrucomicrobiae	Male	0.00157	1
Erysipelotrichia	Male	0.019988	0.91001
Flavobacteriia	Male	0.188945	0.903087
Fusobacteriia	Male	0.194854	0.880107
Acidimicrobiia	Male	0.023936	0.76116
Deltaproteobacteria	Male	0.030305	0.683191
Phycisphaerae	Male	0.024025	0.653271
Spartobacteria	Male	0.003799	0.612415
Mollicutes	Male	0.000428	0.596144
Saccharibacteria;NA*	Male	0.050121	0.594187
Anaerolineae	Male	0.007044	0.583873
Cytophagia	Male	0.031661	0.577974
Spirochaetes	Male	0.006364	0.560617
Chloroflexia	Male	0.012122	0.550016
Negativicutes	Male	0.118259	0.5064

Table S7. Exploratory time-associated candidate taxa identified from mean relative abundance patterns across T1, T96, and T192 samples. Specificity represents the proportion of total abundance attributable to the aging interval exhibiting the highest mean abundance. These taxa are presented as candidate biomarkers of microbial succession during LF aging. (*) depicts taxa detected in one donor only.

Taxon	Time Point	Max Abundance	Specificity
Chlorobia	T1	0.001	1.000
Dehalococcoidia	T1	0.028	1.000
Nitrospira	T1	0.004	1.000
Planctomycetes;c__NA	T1	0.015	1.000
Spirochaetes	T1	0.015	0.879
Epsilonproteobacteria	T1	0.024	0.830
Verrucomicrobiae	T1	0.002	0.786
Thermomicrobia	T1	0.018	0.689
Cytophagia	T1	0.050	0.610
Flavobacteriia	T1	0.185	0.591
Deinococci	T1	0.021	0.568
Gemmatimonadetes	T1	0.002	0.537
Saccharibacteria;NA*	T1	0.063	0.500
Negativicutes	T1	0.166	0.474
Chloroflexia	T1	0.015	0.450
Gracilibacteria*	T1	0.002	0.438
Actinobacteria;c__NA	T96	0.002	1.000
Nitriliruptoria	T96	0.002	1.000
Proteobacteria*	T96	0.007	1.000
Verrucomicrobia*	T96	0.002	1.000
Thermoleophilia	T96	0.130	0.764
Mollicutes	T96	0.001	0.596
Sphingobacteriia	T96	0.183	0.562
Acidimicrobiia	T96	0.026	0.559
Anaerolineae	T96	0.009	0.509
Fusobacteriia	T96	0.161	0.485
Armatimonadetes;c__NA	T192	0.003	1.000
Chlamydiae	T192	0.003	1.000
Spartobacteria	T192	0.009	0.942
Acidobacteria	T192	0.232	0.872
Erysipelotrichia	T192	0.022	0.683
Phycisphaerae	T192	0.037	0.676
Deltaproteobacteria	T192	0.044	0.668
Planctomycetacia	T192	0.067	0.623
Parcubacteria;NA*	T192	0.157	0.620
Coriobacteriia	T192	0.104	0.475
